# Merkel cell polyomavirus large T antigen binding to pRb promotes skin hyperplasia and tumor development

**DOI:** 10.1371/journal.ppat.1010551

**Published:** 2022-05-13

**Authors:** Megan E. Spurgeon, Jingwei Cheng, Ella Ward-Shaw, Frederick A. Dick, James A. DeCaprio, Paul F. Lambert

**Affiliations:** 1 McArdle Laboratory for Cancer Research, Department of Oncology, University of Wisconsin School of Medicine and Public Health, Madison, Wisconsin, United States of America; 2 Department of Molecular, Cellular and Biomedical Sciences, University of New Hampshire, Durham, New Hampshire, United States of America; 3 Department of Pathology and Laboratory Medicine, Western University, London, Ontario, Canada; 4 Children’s Health Research Institute, London, Ontario, Canada; 5 London Regional Cancer Program, London Health Sciences Centre, London, Ontario, Canada; 6 Department of Medical Oncology, Dana-Farber Cancer Institute, Boston, Massachusetts, United States of America; 7 Department of Medicine, Brigham and Women’s Hospital, Harvard Medical School, Boston, Massachusetts, United States of America; Brown University, UNITED STATES

## Abstract

Clear evidence supports a causal link between Merkel cell polyomavirus (MCPyV) and the highly aggressive human skin cancer called Merkel cell carcinoma (MCC). Integration of viral DNA into the human genome facilitates continued expression of the MCPyV small tumor (ST) and large tumor (LT) antigens in virus-positive MCCs. In MCC tumors, MCPyV LT is truncated in a manner that renders the virus unable to replicate yet preserves the LXCXE motif that facilitates its binding to and inactivation of the retinoblastoma tumor suppressor protein (pRb). We previously developed a MCPyV transgenic mouse model in which MCC tumor-derived ST and truncated LT expression were targeted to the stratified epithelium of the skin, causing epithelial hyperplasia, increased proliferation, and spontaneous tumorigenesis. We sought to determine if any of these phenotypes required the association between the truncated MCPyV LT and pRb. Mice were generated in which K14-driven MCPyV ST/LT were expressed in the context of a homozygous Rb^ΔLXCXE^ knock-in allele that attenuates LT-pRb interactions through LT’s LXCXE motif. We found that many of the phenotypes including tumorigenesis that develop in the K14-driven MCPyV transgenic mice were dependent upon LT’s LXCXE-dependent interaction with pRb. These findings highlight the importance of the MCPyV LT-pRb interaction in an *in vivo* model for MCPyV-induced tumorigenesis.

## Author summary

Merkel cell polyomavirus (MCPyV) causes a highly aggressive form of skin cancer called Merkel cell carcinoma (MCC). In human MCC tumors, two viral proteins are expressed: truncated large T antigen (LT) and small T antigen (ST). It is hypothesized that the interaction between MCPyV LT and a cellular tumor suppressor protein, the retinoblastoma protein (pRb), is involved in MCPyV-associated MCC development. In this study, we found that the LT-pRb interaction promotes hyperplasia, proliferation, and tumor development in the skin of transgenic mice expressing the MCPyV LT and ST antigens. This is the first study to directly interrogate a function of the MCPyV LT in skin and highlights the importance of its specific interaction with the host protein pRb in promoting cutaneous phenotypes. These results not only provide insight into the mechanisms of the individual T antigen oncoproteins that are potentially important during virus-induced human disease, but also advance our understanding of how functions of the MCPyV T antigens potentiate changes in the skin, a proposed site of MCPyV tissue tropism.

## Introduction

Merkel cell carcinoma (MCC) is a rare and highly aggressive human cancer associated with several risk factors, including advanced age, light-colored skin, ultraviolet exposure, and immunosuppression [1–3]. In the United States, the incidence of MCC has been predicted to rise in the coming years due to the growing aged population [4]. Merkel cell polyomavirus (MCPyV) was discovered in 2008 when Feng and colleagues identified a polyomavirus clonally integrated in MCC [5]. To date, MCPyV is the only known human polyomavirus causally associated with a human malignancy [6,7]. MCPyV infection is ubiquitous in the human population, largely asymptomatic, and likely occurs during early childhood [8–15]. Because MCPyV is associated with a skin malignancy, can be detected in skin swabs of healthy individuals [16], and can infect and replicate in both keratinocytes and dermal fibroblasts [17–21], the prevailing hypothesis is that MCPyV causes MCC through its infection of cells that normally reside in the skin; however, the cell of origin for MCC has not yet been identified.

In MCPyV-positive MCC tumors, sequences from the early region of the viral DNA genome are clonally integrated into a host cell chromosome, leading to expression of the viral small T (ST) antigen and a truncated form of the viral large T (LT) antigen [5]. The integrated MCPyV T antigens have been implicated in transformation and tumorigenesis in both *in vitro* and *in vivo* studies (Reviewed in [7,22,23]). We previously reported that these viral early proteins, together, act as tumor promoters in the skin of *K14Cre-MCPyV168* transgenic mice [24]. The importance of the T antigens in MCC tumorigenesis is underscored by the fact that their expression is required for optimal MCC cell growth and proliferation [25–27]. Evidence suggests that ST is more dominant than LT in promoting transformation in standard transformation assays: either alone or in combination with LT, the MCPyV ST protein can transform rodent and human fibroblasts *in vitro* [25, 28–32], which correlates with its ability to cause hyperphosphorylation of the translation initiation factor eIF4E binding protein 1 (4E-BP1) [31,33]. In MCC cell lines, ST can specifically recruit MYCL and MAX to the EP400 complex to transactivate a large number of genes that contribute to its transforming functions [34]. MCPyV ST, alone, has also been shown to be tumorigenic in transgenic mouse models [31,35–37] and promotes the development of intra-epidermal MCC-like lesions when expressed together with the Merkel cell specification factor atonal homolog 1 (Atoh1) in epithelial cells [36]. More recently, Verhaegen and colleagues have demonstrated the development of MCC with striking phenotypic and molecular similarities to human MCC in mice expressing MCPyV LT and ST together with Atoh1 on a p53-deficient background [35]. On the other hand, evidence for a robust transforming activity of MCPyV LT has not yet been demonstrated [25,30]. Nevertheless, MCC tumor cell proliferation is reliant on LT expression and function [38,39], suggesting a critical role for MCPyV LT in MCC.

In MCC tumors, integration of the MCPyV genome invariably leads to truncating mutations within the C-terminus of LT [40–42]. The resulting truncated LT proteins typically lack LT’s DNA binding domain and helicase activity, thus preventing viral DNA replication in MCC tumors. However, the truncated LT retains the LXCXE motif within the N-terminus that mediates its interaction with the retinoblastoma protein (pRb) [28,42,43], a member of the pocket protein family of cellular tumor suppressors. Pocket proteins are targeted for inactivation by many oncogenic viruses, leading to cell cycle deregulation and cellular proliferation [44]. The LXCXE motif in the pRb-binding domain of MCPyV LT is required for its interaction with pRb, and mutagenesis of the LXCXE motif abolishes LT’s ability to co-immunoprecipitate pRb-LT complexes *in vitro* [42]. Several lines of evidence indicate that LT-mediated inactivation of pRb is critical to its ability to promote MCC tumor cell growth and proliferation [25,38,39,45,46]. In a human MCC xenograft model, MCC xenografts regressed when LT expression was silenced and the LXCXE-dependent interaction of LT and pRb was found to be necessary for MCC tumor growth [39]. In immortalized fibroblasts, the association of LT with pRb is critical for its ability to promote cellular proliferation and motility [47]. Sequencing of MCC tumors reveals that MCPyV-negative MCC tumors typically contain mutations that inactivate the *RB1* gene; whereas most MCPyV-positive MCCs contain wildtype *RB1* gene [46, 48]. MCPyV-positive MCCs also have a lower number of somatic mutations, including other tumor suppressors such as p53, than do MCPyV-negative MCCs. Unlike SV40 LT, which inactivates both pRb and p53, we have shown that MCPyV LT activates p53 by upregulating expression of ARF, which inhibits MDM2, a protein that induces degradation of p53 [49]. In the same study, we demonstrated that co-expression of MCPyV ST reduced p53 activity through activation of MDM2 [49]. Together, these findings support the hypothesis that tumor suppressor inactivation including pRb is key to MCPyV-driven MCC. Recent studies indicate that truncated LT promotes trans-differentiation of MCC precursor cells, at least in part by increasing expression of Atoh1 in an LXCXE-dependent manner [50,51]. Therefore, it is clear that truncated LT and its association with pRb is important in promoting MCC cell growth and proliferation.

We have previously developed a MCPyV transgenic mouse model (*K14Cre-MCPyV168*), in which expression of both truncated LT and intact ST, as expressed in MCC tumors, is directed to the skin using a keratin 14 (K14) promoter. Their expression led to hyperproliferation, increased expression of MCM7, an E2F-responsive gene, and spontaneous epithelial tumorigenesis in the skin [52]. Because the MCPyV LT-pRb interaction is predicted to increase E2F activity, we sought to determine if the ability of MCPyV to interact with pRb contributes to acute phenotypes and adult-onset tumorigenesis in our *K14Cre-MCPyV168* transgenic mice. To do so, we made use of *Rb*^*ΔLXCXE*^ (or *Rb*^*ΔL*^) knock-in mice, in which three residues within the pRb cleft required for binding LXCXE containing peptides were mutated, rendering the pRb protein incapable of binding LXCXE motif-containing proteins including adenovirus E1A and high-risk human papillomavirus (HPV) E7 oncoproteins, but retains other functions of pRb such as its ability to bind and repress E2F transcription factors [53–55]. We have previously used the same *Rb*^*ΔL*^ mice to explore pRb-dependent and pRb-independent functions of another viral protein, HPV16 E7, *in vivo* [56,57]. Here, we generated *K14Cre-MCPyV168-Rb*^*ΔL*^ mice to determine the specific contribution of the LXCXE-dependent interaction between MCPyV LT antigen and pRb protein to epithelial phenotypes and tumorigenesis observed in *K14Cre-MCPyV168* transgenic mice.

## Results

### The Rb^ΔLXCXE^ mutation in pRb reduces its interaction with MCPyV LT

**Fig 1:**
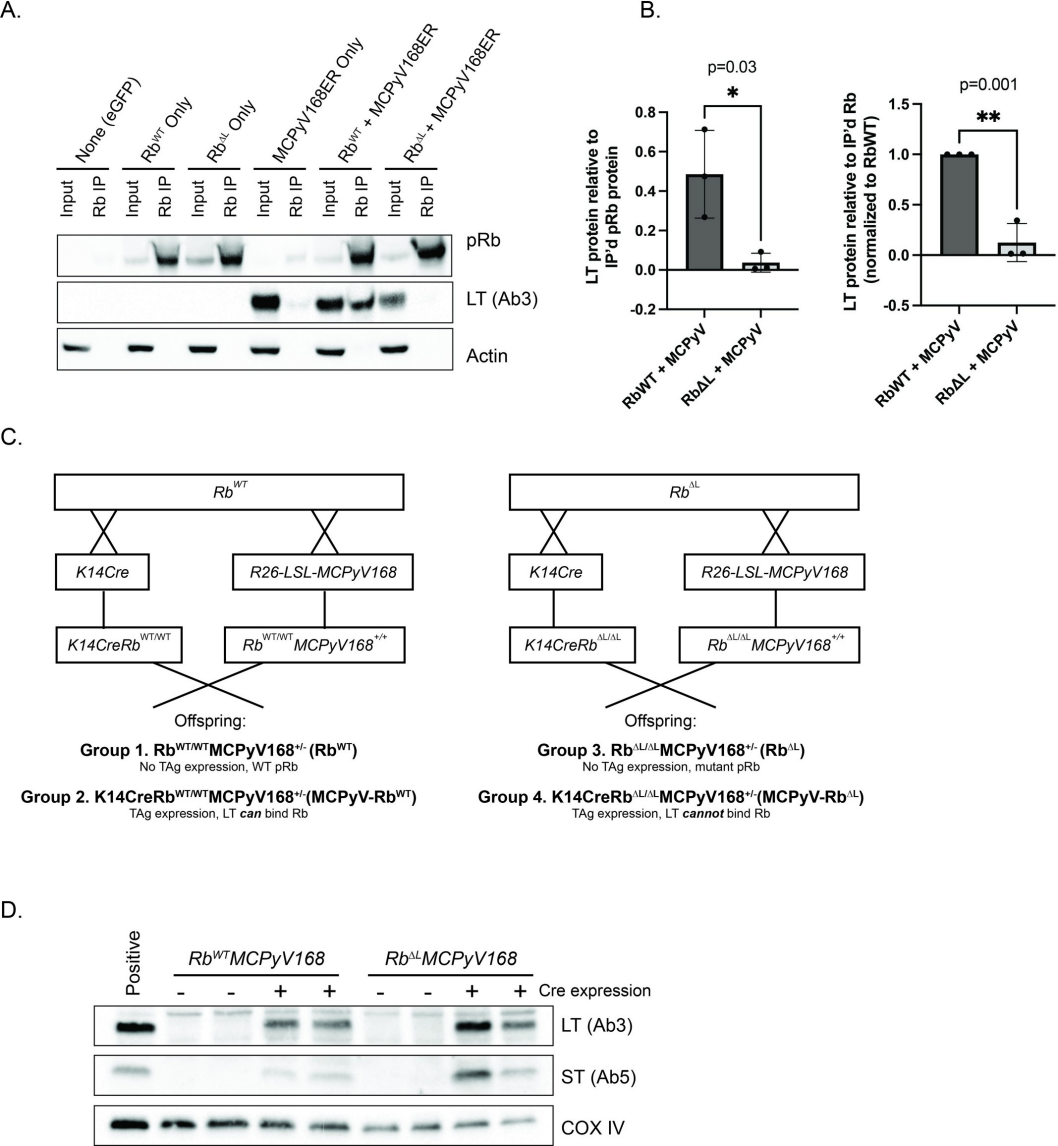
The Rb^ΔL^ mutation in pRb disrupts its interaction with MCPyV LT but does not affect K14-directed expression of MCPyV T antigens in the skin of mice. **A)** Co-immunoprecipitation and immunoblot analysis of the MCPyV large T antigen (LT) and pRb. 293FT cells were transfected with pcDNA-dsRed plasmid (none), pRb^WT^ only, pRb^ΔL^ only, a MCPyV-168ER expression construct expressing the MCPyV168 early region only, or co-transfected with pRb^WT^ + MCPyV168ER or pRb^ΔL^ + MCPyV168ER. At 48 hours post-transfection, whole cell lysates were collected. Using an anti-pRb antibody, the pRb protein was immunoprecipitated from a total of 1 mg of total protein lysate. Pre-cleared lysate corresponding to approximately 3% (30 μg) of that used in the immunoprecipitation assay was used as an input control. The input controls and immunoprecipitated complexes were then analyzed by western blotting for pRb (top), the MCPyV LT antigen (middle; Ab3 antibody) and β-actin as a loading control. **B)** Quantitation of co-immunoprecipitated MCPyV LT protein normalized to the amount of immunoprecipitated pRb protein. Three independent replicates of pRb immunoprecipitation followed by pRb and MCPyV LT immunoblotting were performed. Using densitometry, the total amount of immunoprecipitated pRb and MCPyV LT co-immunoprecipitated with pRb was quantified. The results are shown as direct values (left) or normalized to pRb-WT (right). Error bars indicate standard deviation. Statistical comparisons were performed using unpaired t-tests and p-values are indicated. Please refer to the text for more information regarding statistical comparisons. **C)** Overview of breeding scheme to generate the four experimental groups of mice included in this study. All groups of mice were generated to remain homozygous for either the Rb^WT^ or Rb^ΔLXCXE^ (Rb^ΔL^) alleles. For each group of mice, the abbreviated genotype label is indicated in parentheses. **D)** Immunoblot analysis of whole skin lysates isolated from *Rb*^*WT*^*MCPyV168* and *Rb*^*ΔL*^*MCPyV168* transgenic mice alone (-Cre) or crossed with *K14Cre* (+Cre). Skin lysates (50 μg total protein) were analyzed for MCPyV large T (LT) expression with the Ab3 antibody, small T antigen expression (ST) with the Ab5 antibody, and COX IV as a loading control.

We have previously shown that epithelial expression of the MCPyV T antigens in murine skin leads to the development of epithelial hyperplasia, increased proliferation, and spontaneous tumorigenesis, as well as strong induction of E2F-dependent gene expression indicative of LT’s inactivation of the pRb protein [52]. We therefore sought to determine the specific role of MCPyV LT’s interaction with pRb in the development of epithelial phenotypes and tumorigenesis in *K14Cre-MCPyV168* MCPyV transgenic mice. To do so, we utilized a knock-in mouse strain (*Rb*^Δ*LXCXE*^ or *Rb*^*ΔL*^*)* that expresses a mutant form of pRb containing three alanine substitutions in the binding cleft that renders the protein unable to bind proteins with the LXCXE motif such as HPV16 E7 [53,54]. The *Rb*^*ΔLXCXE*^ mutant retains binding to cellular E2Fs, induces G1 arrest in pRb-null cells, and is regulated by cyclin D/cdk4 complexes similarly to wild-type pRb [54]. To verify that the mutations present in the mutant Rb^ΔL^ protein specifically prevent binding to MCPyV LT, we overexpressed either the wild-type pRb or the mutant pRb^ΔL^ protein alone, or with a construct containing the same MCPyV early region DNA sequence expressed in our transgenic mice (MCCw168 early region), in 293FT cells. Using whole cell lysates collected at 48 hours post-transfection from three independent experiments, we immunoprecipitated pRb and performed a western blot for the truncated version of MCPyV LT (Fig 1A). We used densitometry to quantify the amount of LT immunoprecipitated with either the wild-type pRb or the mutant pRb^ΔL^ protein (Fig 1B). As shown in both the immunoblot and quantified results, we observed specific co-immunoprecipitation of LT and wild-type pRb. There was a significant reduction in the amount of LT immunoprecipitated with the mutant form of pRb compared to wild-type pRb (direct values for LT/immunoprecipitated pRb: 0.01 vs. 0.49, p = 0.03; normalized to RbWT: 0.12 vs. 1.0, p = 0.001; unpaired t-test). However, we still observed a small amount of LT co-immunoprecipitated with Rb^ΔL^. Therefore, the Rb^ΔL^ mutation significantly reduces, but does not completely eliminate, the interaction of Rb with MCPyV LT. Given this result, we used this allele to assess the role of the LT-pRb interaction in promoting epithelial phenotypes in *K14Cre-MCPyV168* mice.

We generated MCPyV transgenic mice on either a homozygous pRb wild-type (WT) or pRb mutant (Rb^ΔL^) background by crossing *R26-LSL-MCPyV168* with either *RbWT* or *Rb*^*ΔL*^ mice. To maintain the pRb status homozygous in all groups, we also generated *K14Cre-Rb*^*WT/WT*^ or *K14Cre- Rb*^*ΔL/ΔL*^ mice. As a result, four groups of mice were included in this study (Fig 1C): 1) *Rb*^*WT/WT*^*MCPyV168*^*+/-*^ (Rb^WT^) mice that have a wild-type pRb protein and no MCPyV T antigen expression in the absence of Cre recombinase, 2) *K14CreRb*^*WT/WT*^*MCPyV168*^*+/-*^ (MCPyV-Rb^WT^) mice that have a wild-type pRb protein and express MCPyV ST and truncated MCPyV LT antigens in K14-positive epithelial cells, 3) *Rb*^*ΔL/ΔL*^*MCPyV168*^*+/-*^ (Rb^ΔL^) mice that express the mutant pRb protein and no MCPyV T antigen expression, and 4) *K14CreRb*^Δ*L/*ΔL^*MCPyV168*^*+/-*^ (MCPyV-Rb^ΔL^) mice that express the MCPyV T antigens in the stratified epithelia on the mutant pRb background. All mice were on a mixed *C57BL/6*:*FVB/N* genetic background. To verify our models, we performed western blots for the MCPyV T antigens using skin lysates prepared from all four groups of mice. As expected, we did not detect T antigen expression in the absence of Cre recombinase in either Rb^WT^ or Rb^ΔL^ mice (Fig 1D). In Cre-positive mice, we observed comparable levels of both ST and truncated LT antigens in the skin of MCPyV-Rb^WT^ and MCPyV-Rb^ΔL^ mice, confirming expression of the MCPyV T antigens in the skin of mice regardless of pRb protein status.

### Overt phenotypes and epithelial hyperplasia are significantly reduced, but not eliminated, in the skin of MCPyV-Rb^ΔL^ mice

**Fig 2:**
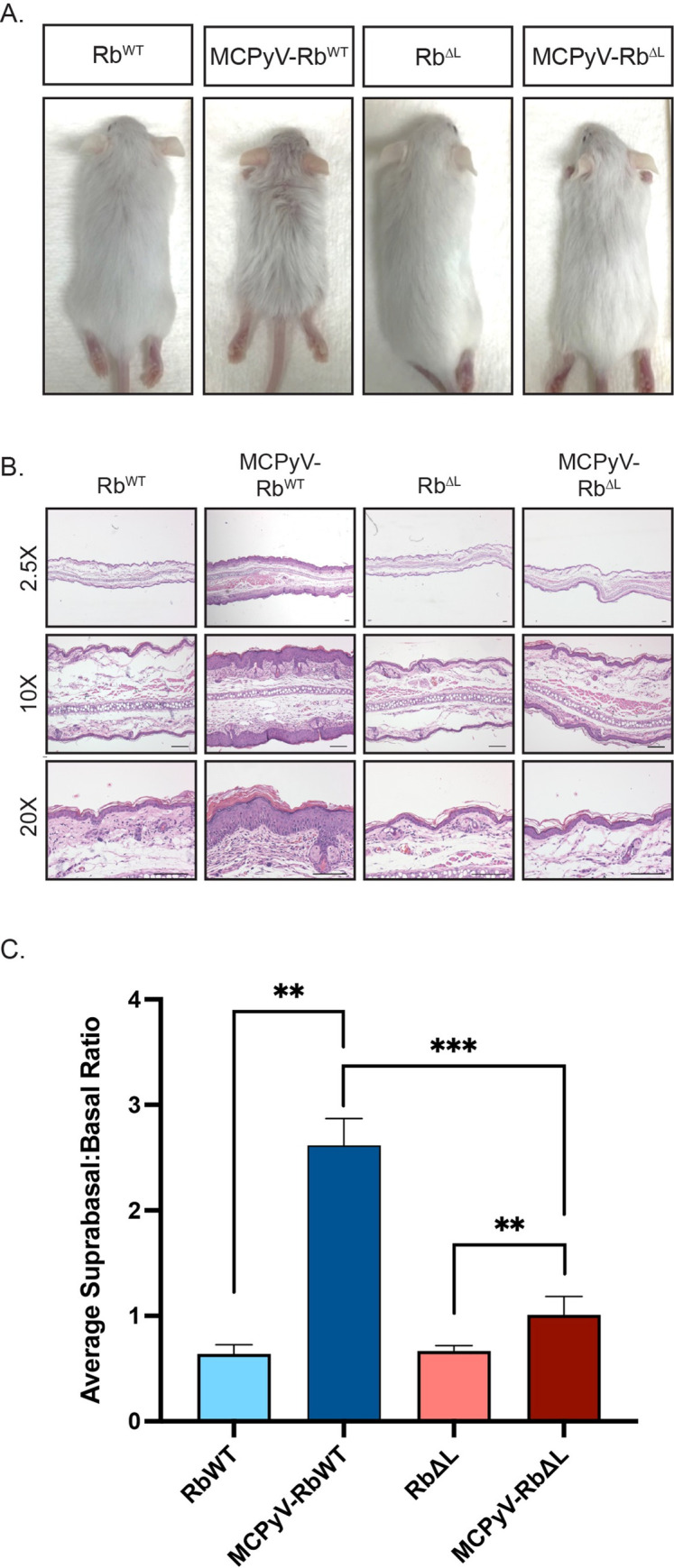
Overt phenotypes and epithelial hyperplasia are significantly reduced, but not eliminated, in the skin of MCPyV-Rb^ΔL^ mice. **A)** Representative images of Rb^WT^, MCPyV-Rb^WT^ Rb^ΔL^, and MCPyV-Rb^ΔL^ mice on a mixed genetic background at approximately 3 weeks of age. The mixed *C57BL/6* and *FVB/N* genetic background yielded both brown and white offspring. Phenotypes were more visually apparent in white offspring, which are shown in this figure. All photographs were taken at the same distance from each subject and therefore reflect the same magnitude. **B)** Representative images of H&E-stained sections of ear tissue harvested from Rb^WT^, MCPyV-Rb^WT^ Rb^ΔL^, and MCPyV-Rb^ΔL^ mice are shown at low-magnification (2.5X; top) and higher magnifications (10X; middle, 20X; bottom). All scale bars = 100 μm. C) Quantification of epithelial hyperplasia in ear tissues harvested from Rb^WT^, MCPyV-Rb^WT^ Rb^ΔL^, and MCPyV-Rb^ΔL^ mice. Epithelial hyperplasia was measured by counting the total number of cells, the number of basal cells, and the number of suprabasal cells present in the stratified squamous epithelium present in a 10X field of view for each sample. The total number of suprabasal cells were then divided by the total number of basal cells to calculate the suprabasal:basal ratio. Ten-10X images were captured for each sample, analyzed using the process outlined above, and an average suprabasal:basal ratio calculated. Within each group, 5–8 mice were included and an average of the averages are plotted in the bar graph. Error bars represent standard deviation. A Wilcoxon Rank Sum test was used to compare values among groups. Asterisks indicate statistical significance (**p≤0.002, ***p≤0.0007). Please refer to the text for more information regarding statistical comparisons.

In our previously reported study, we observed that epithelial expression of the MCPyV T antigens induced several gross phenotypes including small body size, ruffled pelage, hyperplastic skin, and acanthosis or skin thickening that was particularly evident on the ears [52]. In a mixed *C57BL/6* and *FVB/N* genetic background, many of these overt phenotypes resolved by approximately 2 months of age. Because our current study involves mice on a mixed genetic background, we assessed 3-week-old weanlings from all four groups of mice for the development of overt phenotypes (Fig 2A). In the absence of MCPyV T antigen expression, both Rb^WT^ and Rb^ΔL^ mice appeared normal. In *K14CreRb*^*WT*^*-MCPyV168* mice expressing the MCPyV T antigens on a wild-type pRb background (MCPyV-Rb^WT^), we observed severe epithelial phenotypes, including small body size, ruffled fur, and thickened skin, consistent with our previous study. Remarkably, these overt phenotypes were almost completely absent in *K14CreRb*^*ΔL*^*-MCPyV168* mice expressing MCPyV T antigens on the Rb^ΔL^ background (MCPyV- Rb^ΔL^), despite the continued expression of MCPyV LT and ST in the skin (Fig 1D). These results indicate that the LXCXE-dependent interaction between truncated MCPyV LT and pRb is a prominent driver of overt phenotypes in our MCPyV transgenic mice.

We harvested ear tissue from 3-week-old mice from all four groups to evaluate the histopathology of the skin. We imaged hematoxylin and eosin (H&E)-stained sections from these samples (Fig 2B) and used the resulting images to quantify epithelial hyperplasia (Fig 2C). The total number of suprabasal and basal cells comprising the full thickness of the stratified epithelium of ear skin was counted in samples harvested from Rb^WT^ (n = 6), MCPyV-Rb^WT^ (n = 6), Rb^ΔL^ (n = 5), and MCPyV-Rb^ΔL^ (n = 8) mice. The number of suprabasal cells were divided by the number of basal cells to calculate the suprabasal:basal ratio and assess the degree of epithelial hyperplasia for each sample. In the absence of MCPyV T antigen expression, there was no significant difference in the suprabasal:basal ratios in Rb^WT^ and Rb^ΔL^ skin (0.64 ± 0.09 vs. 0.67 ± 0.05; p = 0.66), indicating that the *Rb*^*ΔL*^ allele, even in a homozygous state, did not affect epithelial hyperplasia. This observation is consistent with our previous findings that the *Rb*^*ΔL*^ mutation does not affect epithelial thickness [57]. In contrast, we observed a significant increase in epithelial hyperplasia in MCPyV-Rb^WT^ skin expressing the MCPyV T antigens on a wild-type pRb background compared to Rb^WT^ skin (2.62 ± 0.25 vs. 0.64 ± 0.09; p = 0.002), and this difference was readily apparent by histopathological analysis of the tissues. Although less visually pronounced, we also observed that MCPyV T antigen expression on the mutant pRb background (MCPyV-Rb^ΔL^) also induced a significant increase in epithelial hyperplasia compared to Rb^ΔL^ mice (0.67 ± 0.05 vs. 1.01 ± 0.17; p = 0.002). However, the suprabasal:basal ratio was significantly reduced in MCPyV-Rb^ΔL^ skin compared to MCPyV-Rb^WT^ skin (1.01 ± 0.17 vs 2.62 ± 0.25; p = 0.0007). These results indicate that the LXCXE-dependent pRb-LT interaction in murine skin contributes significantly to epithelial hyperplasia.

### MCPyV T antigen-induced cellular proliferation is significantly reduced but not eliminated, whereas E2F-dependent gene expression is mostly retained in the skin of MCPyV-Rb^ΔL^ mice

**Fig 3:**
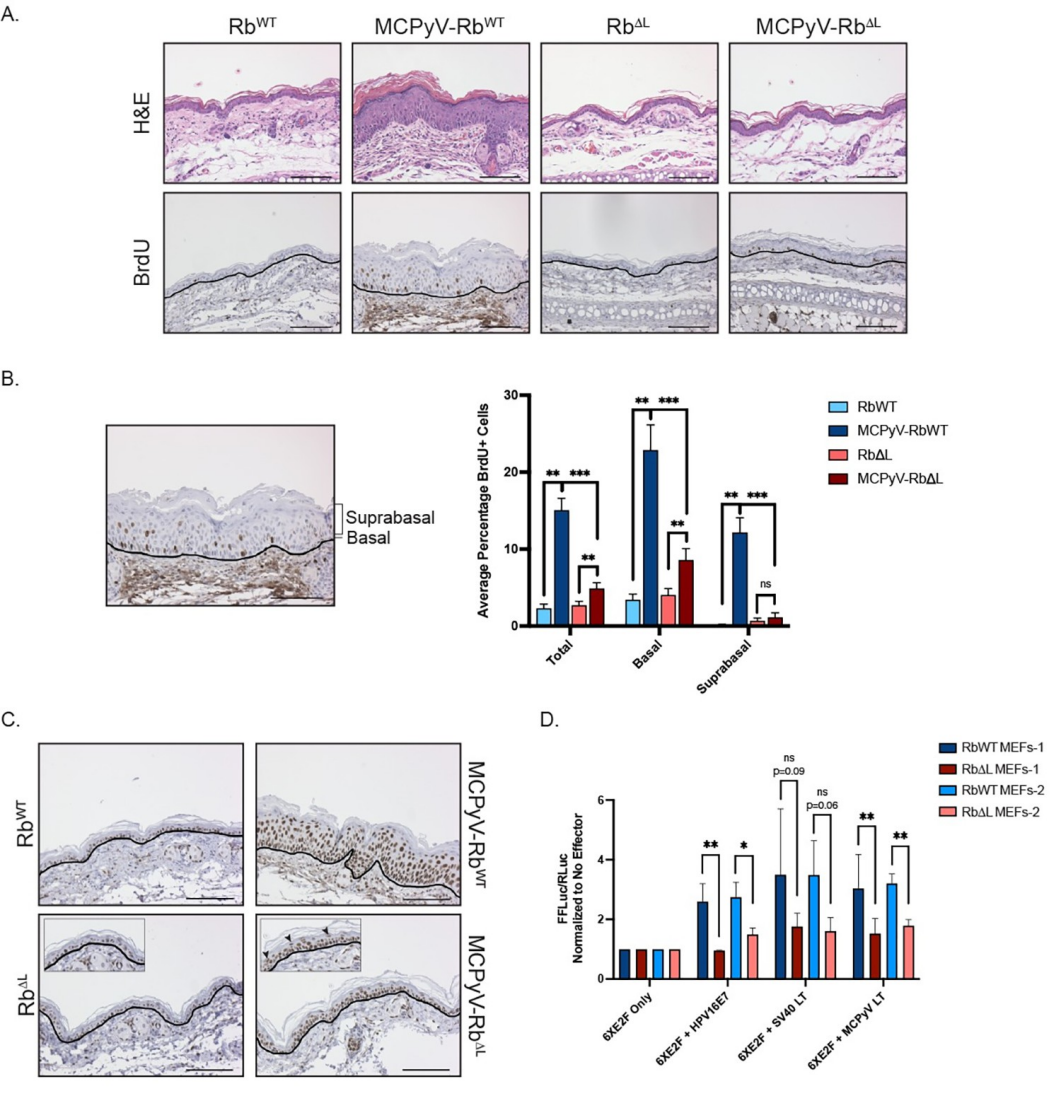
MCPyV T antigen-induced cellular proliferation and E2F-dependent gene expression are significantly reduced, but not eliminated, in the skin of MCPyV-Rb^ΔL^ mice. **A)** Ear tissues harvested from Rb^WT^, MCPyV-Rb^WT^ Rb^ΔL^, and MCPyV-Rb^ΔL^ mice were analyzed using immunohistochemistry for BrdU and counterstained with hematoxylin (bottom). BrdU-positive cells contain brown nuclei. The black line indicates the basement membrane between the epithelium and the underlying dermis. Corresponding representative H&E-stained images are included as a reference (top). All scale bars = 100 μm. **B)** For BrdU quantitation, basal cells were defined as any cell in contact with the basement membrane. Suprabasal cells included all other cells within the stratified epithelium that were not touching the basement membrane. An image is shown with brackets identifying the suprabasal layer and a line pointing to the basal layer. The number of total, basal, and suprabasal BrdU-positive cells in the stratified epithelium were counted in 10 fields of view per slide from each mouse per group. These values were then divided by the total number of epithelial cells in each sample to calculate the percentage of BrdU-positive cells, and these values were then averaged. An average of the averages was then calculated for each group of mice. Error bars indicate standard deviation. A Wilcoxon Rank Sum test was used to compare values among groups. Asterisks indicate statistical significance (**p≤0.002, ***p≤0.0007) and “ns” means non-significant. Please refer to the text for more information regarding statistical comparisons. **C)** Ear tissues harvested from Rb^WT^, MCPyV-Rb^WT^, Rb^ΔL^, and MCPyV-Rb^ΔL^ mice were analyzed using immunohistochemistry for the MCM7 protein and counterstained with hematoxylin (bottom). MCM7-positive cells contain brown nuclei. The black line indicates the basement membrane between the epithelium and the underlying dermis. Insets are enlarged images to highlight suprabasal cells staining positive for MCM7 (black arrowheads). All scale bars = 100 μm. **D)** E2F-luciferase assay results from primary mouse embryonic fibroblasts (MEFs) isolated from pRb^WT^ and pRb^ΔL^ mice transfected with viral protein effectors. MEFs from litter-matched pRb^WT^ and pRb^ΔL^ embryos from two different litters were used: RbWT MEFs-1 and RbΔL MEFs-1 (dark blue and maroon, respectively) and RbWT MEFs-2 and RbΔL MEFs-2 (light blue and pink, respectively). Cells were transfected with an E2F-luciferase reporter construct (6XE2F; Firefly luciferase) only, or co-transfected with 6XE2F reporter plus SV40 large T antigen (LT), the MCPyV LT, or human papillomavirus 16 (HPV16) E7 protein. In all conditions, cells were co-transfected with a control plasmid expressing *Renilla* luciferase that was used to normalize for transfection efficiency. At 48 hours post-transfection, a dual-luciferase assay was performed to quantify firefly and *Renilla* luciferase expression in each condition. Following normalization of all luciferase values (firefly/*Renilla*), the values in cells only transfected with the reporter (6XE2F Only) were set to 1.0 and all other values were compared to this value within each MEFs cell strain. Error bars indicate standard deviation. Unpaired t-tests were used to compare luciferase values between RbWT and RbΔL cells for each condition. Asterisks indicate statistical significance (*p≤0.05, **p≤0.01) and “ns” means non-significant. Please refer to the text for more information regarding statistical comparisons.

In our previous studies, we found that epithelial expression of the MCPyV T antigens significantly increases cellular proliferation as measured by DNA replication via BrdU incorporation [52]. Therefore, we sought to determine the impact of MCPyV T antigen expression on cellular proliferation on a wild-type versus mutant pRb background by injecting mice with BrdU 1 hour prior to sacrifice. Using tissue samples from the same mice used to evaluate epithelial hyperplasia (Fig 2), we performed immunohistochemistry for BrdU on ear tissue sections (Fig 3A) and quantified the average percentage of total, basal, and suprabasal BrdU incorporation (Fig 3B) in all four groups of mice. As expected based on our prior studies [52], in tissues from MCPyV-Rb^WT^ mice compared to Rb^WT^ mice, we observed a significant increase in the average percentage of total (15.0% vs 2.3%; p = 0.002), basal (22.9% vs. 3.4%; p = 0.002), and suprabasal (12.1% vs. 0.08%; p = 0.002) BrdU-positive cells in the stratified epithelium of the ear skin. Likewise, we measured a significant increase in the average percentage of total (4.89% vs. 2.69%; p = 0.0007)) and basal (8.57% vs. 4.01%; p = 0.002) BrdU-positive cells in MCPyV-Rb^ΔL^ tissues compared to Rb^ΔL^ tissues. Interestingly, there was no significant increase in the average percentage of suprabasal BrdU-positive cells in MCPyV-Rb^ΔL^ skin compared to Rb^ΔL^ skin (1.13% vs. 0.65%; p = 0.35). While the MCPyV T antigens appeared to still be capable of increasing the average percentage of total and basal BrdU-positive cells on the Rb^ΔL^ background, it is notable that the overall percentages of total (4.89% vs. 15.04%; p = 0.0007), basal (8.57% vs. 22.87%; p = 0.0007), and suprabasal (1.13% vs. 12.14%; p = 0.0007) BrdU-positive cells in MCPyV-Rb^ΔL^ skin were still significantly lower than those observed in MCPyV-Rb^WT^ mice. These results indicate that the LXCXE-dependent pRb-LT interaction is important to the induction of cellular proliferation in the skin of mice, particularly in the suprabasal layer of the stratified epithelia.

Our previous studies found that K14-directed expression of the MCPyV T antigens significantly increased E2F-dependent gene expression as measured by an E2F-dependent gene, *MCM7* [52]. MCM7 protein expression is normally restricted to the basal cells of the stratified epithelium in wild-type skin. However, MCPyV T antigen expression causes strong MCM7 expression throughout the full thickness of stratified epithelium. Given its nature as an E2F-dependent gene and thus its strong link to pRb inactivation by polyomavirus T antigens, we hypothesized that the induction and reorganization of MCM7 expression was dependent on the MCPyV-LT interaction with pRb. To test this hypothesis, we analyzed the pattern of MCM7 protein expression in ear tissues harvested from Rb^WT^, MCPyV-Rb^WT^, Rb^ΔL^, and MCPyV-Rb^ΔL^ mice using immunohistochemistry (Fig 3C). As expected, MCM7 protein expression was restricted to the basal cell layer of the stratified epithelium in Rb^WT^ mice and we observed a similar pattern of expression in Rb^ΔL^ mice, indicating that the mutations in the pocket domain of Rb^ΔL^ do not affect the MCM7 protein expression pattern. Consistent with our previous observations, there was strong induction and reorganization of MCM7 protein expression in the skin of MCPyV-Rb^WT^ mice. While its intensity was slightly reduced, MCM7 expression was present in basal cells and suprabasal cells throughout the full thickness of the stratified epithelium in MCPyV-Rb^ΔL^ skin (Fig 3C; inset and black arrowheads). These results were somewhat unexpected but may reflect either the residual weak interaction between LT and Rb^ΔL^ that we observed in our co-immunoprecipitation experiments (Fig 1A and 1B) or a heretofore unknown role in E2F-dependent gene expression induced by the continued expression of ST in MCPyV-Rb^ΔL^ mice (Fig 1D).

To quantify the induction of E2F-dependent genes in cells expressing either Rb^WT^ or Rb^ΔL^ and MCPyV LT, we performed *in vitro* luciferase assays. We utilized a reporter containing six E2F consensus sites upstream of the firefly luciferase gene (6XE2F) [58] to measure the ability of MCPyV LT to induce luciferase activity in primary mouse embryonic fibroblasts (MEFs) isolated from matched wild-type or *Rb1*^*ΔL/ΔL*^ littermates [55, 59]. Rb^WT^ and Rb^ΔL^ MEFs from two independent litters were transfected with 6XE2F reporter alone, or co-transfected with a construct expressing the MCPyV truncated LT that is expressed in our MCPyV transgenic mice. We also co-transfected cells with either HPV16 E7 protein or SV40 LT, other viral effector proteins that target the pRb protein for inactivation and thus induce E2F-dependent genes. All cells were also co-transfected with a *Renilla* luciferase construct to serve as a control to normalize for transfection efficiency. At 48 hours post-transfection, dual-luciferase assays were performed to quantify luciferase activity and normalized values were set relative to cells with reporter only. The luciferase activity levels achieved with the 6XE2F reporter in primary MEFs in this study, which are lower than other stronger luciferase reporter constructs, are comparable to levels achieved in other studies using this reporter [60, 61].

In Rb^WT^ MEFs co-transfected with the 6XE2F reporter and MCPyV LT, we observed an approximately 3-fold induction in luciferase over reporter alone, which was significantly reduced in Rb^ΔL^ MEFs (Fig 3D). This reduction was evident in both sets of matched littermate MEF cell strains expressing MCPyV LT (Rb^WT^ vs Rb^ΔL^ MEFs-1 (n = 6): p = 0.01; MEFs-2 (n = 3): p = 0.003; unpaired t-tests). We observed similar reductions in E2F-driven luciferase activity in Rb^ΔL^ MEFs compared Rb^WT^ MEFs expressing HPV16E7 (Rb^WT^ vs Rb^ΔL^ MEFs-1 (n = 3): p = 0.01; MEFs-2 (n = 3): p = 0.02; unpaired t-tests) and SV40 LT (Rb^WT^ vs Rb^ΔL^ MEFs-1 (n = 6): p = 0.09; MEFs-2 (n = 3): p = 0.06; unpaired t-tests) although the reduction in SV40 LT-expressing MEFs did not reach statistical significance. While luciferase activity was significantly reduced in Rb^ΔL^ MEFs expressing MCPyV LT, it was not reduced to the level seen in cells expressing the reporter only. Together, these *in vitro* results indicate that the reduced ability of MCPyV LT to bind the Rb^ΔL^ mutant significantly reduces, but does not completely eliminate, E2F-driven gene expression.

### The LXCXE-dependent interaction between pRb and the MCPyV large T antigen is required for spontaneous tumor development in murine skin

**Fig 4:**
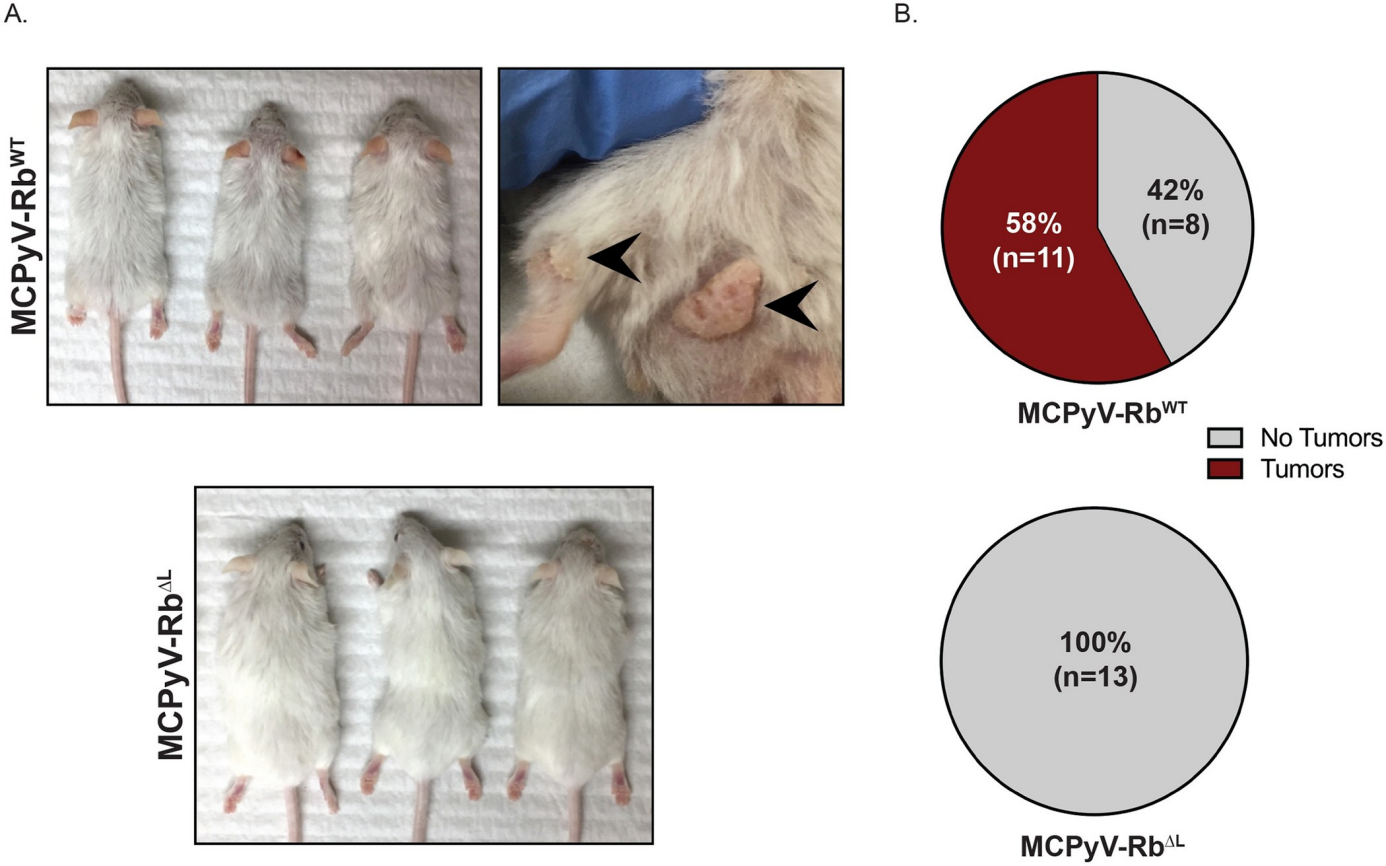
The interaction between pRb and the MCPyV large T antigen is required for spontaneous tumor development in murine skin. **A)** Representative images of MCPyV-Rb^WT^ (top) and MCPyV-Rb^ΔL^ (bottom) mice on the *FVB/N* genetic background at 6 months of age. The black arrowheads highlight two representative spontaneous skin tumors arising on the skin of MCPyV-Rb^WT^ mice. All photographs were taken at the same distance from each subject and therefore reflect the same magnitude. **B)** Pie charts indicate spontaneous tumor incidence in the skin of MCPyV-Rb^WT^ (top) and MCPyV-Rb^ΔL^ (bottom) mice on the *FVB/N* genetic background that developed over the course of 6 months. The number of mice in each condition is shown in parentheses.

We previously reported that *K14Cre-MCPyV168* (same as MCPyV-Rb^WT^ mice in this study) transgenic mice spontaneously develop benign tumors or papillomas on the skin [52]. The development of tumors was only observed in transgenic mice that were maintained on a pure *FVB/N* genetic background. Therefore, to determine the role of LT-pRb interactions in spontaneous skin tumorigenesis, we backcrossed the original *Rb*^*ΔL*^ transgenic mice from the *C57BL/6* genetic background to the *FVB/N* genetic background for at least 10 generations. We then generated the same four groups of mice (Fig 1C) on the *FVB/N* genetic background. Consistent with our previous observations (Fig 2A), the MCPyV-Rb^WT^ mice developed overt epithelial phenotypes that were largely absent in MCPyV-Rb^ΔL^ mice (Fig 4A). As expected based on our prior studies [52], a proportion of MCPyV-RbWT mice developed skin papillomas on the dorsal skin and areas surrounding their hind legs (Fig 4A). Some of these papillomas persisted while some spontaneously regressed, again consistent with our previous observations [52]. We monitored mice over the course of 6 months and counted the number of mice per group that ever developed spontaneous tumors over this time course. Out of 19 total MCPyV-Rb^WT^ mice that were monitored, 58% (n = 11/19) spontaneously developed skin tumors, whereas 42% (n = 8/19) never developed observable or palpable skin tumors (Fig 4B; top) over the 6-month period. Strikingly, all of the MCPyV-Rb^ΔL^ mice (100%; n = 13/13) were tumor-free and never developed skin tumors (Fig 4B; bottom). The difference in tumor incidence between MCPyV-Rb^WT^ and MCPyV-Rb^ΔL^ mice was highly significant (p = 0.0006; two-sided Fisher’s Exact Test). These results indicate that the LXCXE-dependent interaction between pRb and the MCPyV LT antigen is required for spontaneous tumor development in the skin of MCPyV transgenic mice.

## Discussion

Despite strong evidence that the human polyomavirus MCPyV is causally associated with Merkel cell carcinoma (MCC) [22], the relationships between MCPyV and the skin are not yet fully understood. This ongoing area of research has provided insight in recent years, particularly through the use of transgenic mouse models that target expression of those viral proteins implicated in MCC carcinogenesis, truncated LT and ST antigens, to the skin [31,36,37,52]. In the study presented here, we utilized our previously developed MCPyV transgenic mice to explore the role of the LXCXE-dependent interaction between the truncated LT antigen and the retinoblastoma protein (pRb), a host tumor suppressor protein, in the development of cutaneous phenotypes and epithelial tumors. By performing these studies in an *in vivo* system where both MCPyV T antigens are expressed in the skin, we were able to specifically manipulate a function of LT antigen while avoiding disruption of ST functions. We took advantage of a genetically engineered knock-in mouse line that expresses a mutant pRb protein (*Rb*^*ΔLXCXE*^) that is defective in binding to proteins via the LXCXE motif [53–55]. By using the very specific and targeted approach provided by *Rb*^*ΔLXCXE*^ mice, we were able to circumvent the potential pleiotropic and off-target effects associated with germline pRb knockout models and avoid introducing mutations within LT protein that may alter other viral protein functions. Consistent with previous reports, [39,42], we found that the LXCXE motif is required for robust LT-pRb interaction and the mutations present in the mutant Rb^ΔLXCXE^ protein greatly reduce LT binding (Fig 1A and 1B). We discovered that the development of severe overt cutaneous phenotypes (Fig 2A), suprabasal BrdU incorporation indicative of cellular proliferation (Fig 3A and 3B), and spontaneous epithelial tumorigenesis (Fig 4) in MCPyV transgenic mice were dependent on the LXCXE-mediated interaction between truncated LT and pRb. The LT-pRb interaction also significantly contributed to epithelial hyperplasia (Fig 2B and 2C) and basal cell proliferation (Fig 3A and 3B). Interestingly, we still observed a reorganized spatial pattern of E2F-dependent gene expression in the skin of MCPyV transgenic mice on the mutant pRb background (Fig 3C). This study reveals that, in *K14Cre-MCPyV168* mice, the majority of the MCPyV T antigen-induced overt cutaneous phenotypes, histopathological changes, and epithelial tumorigenesis are either exclusively or in large part attributable to the LXCXE-dependent interaction between truncated LT and the tumor suppressor pRb.

This is the first study to directly interrogate a specific function of and identify phenotypes attributable to the MCPyV truncated LT antigen in skin *in vivo*. Various MCPyV transgenic mouse models have been developed that express ST antigen only [31,37], truncated LT only [36], or combined expression of truncated LT and ST [36,52]. In some of these models, ST antigen was identified to be the more potent viral oncoprotein in murine skin. For instance, individual expression of ST is oncogenic in the stratified epithelium of pre-term embryos and adult mice [37] and in the spleen and liver under p53-null conditions [31] and can stimulate Merkel cell proliferation when expressed in Atoh1-positive cells [31]. Moreover, co-expression of ST and Atoh1 in the epithelium was sufficient to facilitate the development of intraepidermal MCC-like lesions in murine skin [36]. More recently, a murine model was reported in which full-blown MCC tumors developed in the skin of mice expressing MCPyV truncated LT and ST together with Atoh1 expression and loss of p53 [35]. Epithelial-specific expression of ST alone also induced many of the same phenotypes we have observed in *K14Cre-MCPyV168* mice expressing ST and LT in the stratified squamous epithelium, including hyperplasia, increased proliferation, and activation of the DNA damage response [31,36,37,52]. However, our findings that most of the phenotypes that develop in our transgenic model are exclusively or in large part driven by LT, even in the presence of continued ST expression, seems to be distinct from results observed by Verhaegen and colleagues. In their report, individual expression of truncated LT did not result in the development of any discernible phenotypes, nor did LT enhance phenotypes in ST mice or ST+Atoh1 mice, leading them to conclude that truncated LT is dispensable for phenotypes and MCC-like tumor development in their model system [36]. There are several possible explanations for these disparate findings regarding the role of LT in transgenic mice. First, it is unlikely these differences are a result of using LT sequences from different MCC tumor-derived MCPyV genomes as transgenes, because both studies used MCPyV DNA derived from the same MCCw168 tumor specimen [28]. One explanation is that Verhaegen *et al*. performed their studies in a panel of pre-term embryos whereas our studies were performed in older mice (3 weeks to 6 months of age). It is possible that certain LT-induced phenotypes develop only after prolonged expression in the skin and were absent in pre-term embryos. Another key difference is that these studies were performed using transgenic mice on either mixed (*FVB/N* and *C57BL/6*; Figs 1–3) or pure *FVB/N* genetic backgrounds (Fig 4) whereas other groups perform studies using mice on pure *C57BL/6* transgenic background. *FVB/N* mice carry a polymorphic variant of the *Ptch1* gene that predisposes them to spontaneous and oncogene-induced squamous carcinoma of the skin [62]. Therefore, it is possible that MCPyV viral oncogenes, and perhaps LT specifically, cooperate with the Hedgehog signaling pathway to induce epithelial phenotypes and tumorigenesis in a unique manner on the *FVB/N* genetic background. Finally, the contrasting results may simply reflect differences between transgenic models in the relative expression of the MCPyV T antigens in the skin due to the different genetic strategies used to target expression.

While many phenotypes in *K14Cre-MCPyV168* transgenic mice were either significantly or entirely reduced on the Rb^ΔL^ background, some of them were not completely eliminated. Epithelial hyperplasia (Fig 2C) and total cell and basal cell proliferation (Fig 3B) were still significantly increased in MCPyV-Rb^ΔL^ mice compared to Rb^ΔL^ mice. We also found that aberrant expression of MCM7, an E2F-dependent gene, was largely retained in suprabasal layers of skin from MCPyV-Rb^ΔL^ mice (Fig 3C). There are several potential explanations for these persistent phenotypes in the absence of robust LT-pRb binding. The most straightforward explanation is that they result from the retained, albeit weak, binding between Rb^ΔL^ and LT (Fig 1A). The mutations present in Rb^ΔL^ were identified using the crystal structure of an HPV E7 peptide containing its LXCXE motif bound to pRb, and therefore were designed to specifically eliminate binding of pRb to E7 [54]. Subsequent studies by Dick *et al*. identified other residues outside of the LXCXE binding pocket that contribute to pRb-E7 interactions, including a conserved lysine patch that, when mutated, not only disrupted the interaction between pRb and E7, but also disrupted the interaction with the LT from another polyomavirus, SV40 [53]. Another group independently and similarly found that this lysine patch contributes to the interaction between pRb and SV40 LT [63]. It is possible that MCPyV LT is more reliant upon these additional residues outside of the LXCXE binding pocket than either HPV E7 or adenovirus E1A. While it is unclear whether MCPyV LT interacts with pRb in the exact manner as SV40 LT, these observations suggest that there are regions outside of the LXCXE binding cleft of pRb that could allow some level of continued interaction between MCPyV LT and the pRb^ΔL^ mutant and allow phenotype development and suprabasal E2F-dependent gene expression in MCPyV-Rb^ΔL^ mice. Considering this evidence, it is worth noting that our E2F luciferase assay results (Fig 3D) showed, albeit *in vitro*, that a continued low level of E2F-dependent gene expression occurs in pRb^ΔL^ MEFs expressing MCPyV LT, as well as SV40 LT and HPV16 E7. It is also possible that other E2F regulators are involved. Although it has been reported that MCPyV LT only binds pRb and not the other pocket proteins p107 and p130 [38], it is possible that LT interacts with the other pocket proteins under different conditions or *in vivo*.

There are other plausible explanations for the residual phenotypes in MCPyV-Rb^ΔL^ mice, including the continued co-expression and function of ST. Both hyperplasia and increased proliferation are well-documented phenotypes associated with epithelial ST expression in murine skin [31,37]. However, it is notable that the LT-pRb binding in MCPyV-Rb^WT^ mice significantly increased both hyperplasia and proliferation compared to MCPyV-Rb^ΔL^ where LT-pRb binding is reduced but ST expression is unaffected, indicating that ST function plays a minor role in promoting these phenotypes in this model. While our current results suggest the LXCXE-dependent interaction with pRb is the predominant function associated with the ability of LT to induce phenotypes in murine skin, the interaction of LT with other cellular proteins may also contribute to phenotypes in *K14Cre-MCPyV168* mice. For instance, LT binds to USP7 [64] and Vam6p [65], and although these interactions were proposed to contribute to viral replication, it is possible they may also contribute to oncogenic development *in vivo*. There are several other additional functions and domains of LT [66] that may also play a role. Finally, it is possible that the residual phenotypes in MCPyV-Rb^ΔL^ mice arise from the effects of the *Rb*^*ΔL*^ mutation itself, which have been reported to include disruptions to heterochromatin structure that lead to aneuploidy and genomic instability [55]. However, we find this to be unlikely as we did not observe similar residual phenotypes in Rb^ΔL^ mice in the absence of MCPyV T antigens.

Our finding that the LXCXE-dependent interaction between truncated LT and pRb is a significant contributor to many of the T antigen-induced overt and molecular phenotypes that develop in the skin of *K14Cre-MCPyV168* transgenic mice is consistent with the documented importance of MCPyV LT in MCC biology. *In vitro*, LT is more important than ST in the maintenance of MCC cell proliferation and survival [25] and this ability to promote MCC cell growth is dependent on an intact LXCXE Rb-binding motif [39]. These findings were replicated in immortalized human fibroblasts, where Richards *et al*. found that truncated LT promotes proliferation in an LXCXE-dependent manner [47]. These LT functions are aligned with our results *in vivo* showing that epithelial hyperplasia (Fig 2), increased epithelial cell proliferation (Fig 3), and spontaneous tumorigenesis (Fig 4) are largely or completely dependent on the LXCXE-dependent interaction between LT and pRb in murine skin. Binding of LT to pRb via the LXCXE motif also contributes to the vast majority of LT-induced gene expression changes in human fibroblasts *in vitro* [47], and it would be interesting to explore whether the same is true in epithelial cells of the skin *in vivo*. In addition to its role in promoting proliferation and broad changes in host gene expression, there is growing evidence that LT is involved in the induction of key Merkel cell lineage genes *in vitro*. Expression of MCPyV LT and ST in keratinocytes increases early Merkel cell markers keratin 8 and keratin 18 [67] and additional studies have more specifically implicated LT as the viral protein responsible for inducing Merkel cell-associated genes. Expression of LT increases *ATOH1* gene transcription in fibroblasts [50] and also stabilizes Atoh1 protein levels in HEK293 and U2OS cells [67]. Furthermore, truncated LT increases Sox2 expression in an LXCXE-dependent manner, and this in turn increases Atoh1 expression in keratinocytes [51]. While this collection of *in vitro* evidence is compelling, we have yet to observe conclusive results demonstrating that epithelial expression of the MCPyV T antigens increases Merkel cell markers in *K14Cre-MCPyV168* transgenic skin tissue samples. Notably, epithelial-targeted expression of ST and/or LT antigens also failed to induce the Merkel cell markers K8 and Sox2 in other transgenic models in the absence of Atoh1 co-expression [36]. It is possible that the mechanisms underlying T antigen mediated induction of Merkel cell-related gene expression *in vivo* differ from those *in vitro*, or there are factors present in human but not mouse cells that render the MCPyV T antigens sufficient to induce Merkel cell gene expression in keratinocytes. Additional studies are warranted to explore these differences.

In conclusion, our results illuminate the importance of the specific interaction between the MCPyV LT antigen and the pRb tumor suppressor protein in promoting cutaneous phenotypes in an *in vivo* transgenic model of MCPyV T antigen expression. This is the first study to directly interrogate a function of the MCPyV LT in skin in an *in vivo* context and our results provide important insight into functions of the individual T antigens in a proposed site of MCPyV tissue tropism. Future studies utilizing both existing and continuously evolving transgenic mouse models of MCPyV T antigen expression provide exceptional opportunities to further characterize the relationship between these proposed viral oncoproteins, the skin, and virus-induced disease.

## Materials and methods

### Ethics statement

All animal experiments were performed in full compliance with standards outlined in the Guide for the Care and Use of Laboratory Animals by the Laboratory Animal Resources (LAR) as specified by the Animal Welfare Act (AWA) and Office of Laboratory Animal Welfare (OLAW) and approved by the Governing Board of the National Research Council (NRC). Mice were housed at the McArdle Laboratory Animal Care Unit in strict accordance with guidelines approved by the Association for Assessment of Laboratory Animal Care (AALAC), at the University of Wisconsin Medical School. All protocols for animal work were approved by the University of Wisconsin Medical School Institutional Animal Care and Use Committee (IACUC; approved protocol number M005871).

### Transgenic mice

The *ROSA26-LSL-MCPyV168* transgenic mice have been described previously [52]. Briefly, these transgenic mice contain a conditional allele in which the MCPyV early region, isolated from MCC tumor specimen MCCw168 (MCPyV168; GenBank: KC426954.1), was cloned downstream of a region containing the triple SV40 polyadenylation sequences that function as transcription terminators (stop) flanked by two loxP sites (loxP-stop-loxP, or LSL). The LSL, MCPyV168, and a bovine growth hormone polyadenylation terminator was cloned into the pROSA26PA plasmid. To express the MCPyV T antigens in the skin, *ROSA26-LSL-MCPyV168* were crossed with transgenic mice expressing Cre recombinase driven by the human keratin 14 (*Krt14* or K14) promoter (*K14Cre*). Both *ROSA26-LSL-MCPyV168* and *K14Cre* mice were maintained on the *FVB/N* murine genetic background. The *Rb*^*ΔLXCXE*^ (also known as *Rb*^*ΔL*^*)* mice have been previously described [55]. The *Rb*^*ΔL*^ transgenic mice contain a knock-in allele that contains 3 alanine mutations in the murine retinoblastoma protein (pRb) binding cleft (I746A, N750A, M754A) that are equivalent to the residues in human pRb shown to block LXCXE-mediated interactions (I753A, N757A, M761A) [54]. The *Rb*^*ΔL*^ mice were maintained on a mixed *C57BL/6* and *FVB/N* genetic background. All studies except tumorigenesis studies were performed on a mixed genetic background with all experimental groups being bred to contain the same level of genetic heterogeneity of *FVB/N* and *C57BL/6* genetic backgrounds. To perform tumorigenesis studies, the *Rb*^*ΔL*^ mice were backcrossed onto the *FVB/N* genetic background for at least 10 generations and then used to regenerate the four groups of mice indicated in Fig 1C.

### Genotyping

All transgenic mice used in these studies were verified by PCR genotyping. Genomic DNA was isolated from tail snips and resuspended in water. Separate PCR reactions were used to identify the wild-type or recombined ROSA26 allele, presence of the Cre recombinase gene, and the Rb^ΔL^ transgenes. PCR products were evaluated using agarose gel electrophoresis. The following primers were used for genotyping: P1 (5’-AAA GTC GCT CTG AGT TGT TAT-3’), P2 (5’-GCG AAG AGT TTG TCC TCA-3’) and P3 (5’-AGC GGG AGA AAT GGA TAT-3’) specific for the ROSA26 allele; 3069 (5’-TTC CTC AGG AGT GTC TTC GC-3’) and 3070 (5’-GTC CAT GTC CTT CCT GAA GC-3’) for K14Cre; FD134 (5’-AGC TTC ATA CAG ATA GTT GGG-3’) and FD135 (5’-CAC ACA AAT CCC CAT ACC TAT G-3’) for Rb^ΔL^. All primers were synthesized by Integrated DNA Technologies (Coralville, IA).

### Tissue procurement/processing

One hour prior to sacrifice, mice were treated with 100mg/kg 5-bromodeoxyuridine (BrdU) by intraperitoneal injection. Mice were euthanized using CO_2_ asphyxiation followed by cervical dislocation and expiration was confirmed by verifying respiratory arrest. Ear tissue was harvested and fixed in 4% paraformaldehyde. Tissues were then histologically processed, paraffin-embedded and serial section at 5μm thickness. Every 10th section was stained with hematoxylin and eosin (H&E).

### Immunoprecipitation and immunoblot analysis

The 293FT cells were maintained in DMEM supplemented with 10% FBS and 1% penicillin/streptomycin. For immunoprecipitation assays, 293FT cells were plated at a density of 1.5 x 10^5^ cells/mL in 10 cm dishes approximately 24 hours prior to transfection in culture media without antibiotics. For overexpressing pRb *in vitro*, pCMV promoter expression constructs pFAD102 (RbWT) and pFAD139 (Rb^ΔLXCXE^) that contain the wild-type pRb or mutant (I753A, N757A, M761A) human pRb cDNA, respectively, have been described previously [54]. To overexpress the MCPyV T antigens, we used the mammalian expression vector pLenti6.3-MCPyV168ER, a construct that contains the same DNA sequence of the MCPyV early region expressed in *Rosa26-LSL-MCPyV168* transgenic mice that was PCR amplified and introduced into the pLenti6.3 lentiviral expression vector (Thermo Fisher; Waltham, MA) via gateway cloning. Using the manufacturer’s protocol, 20 μg of DNA was transfected in single-construct conditions and 24 μg (12 μg of each plasmid) total DNA transfected in co-transfections using Lipofectamine 2000 Transfection Reagent (Thermo Fisher; Waltham, MA). One condition included transfection of a pCMV-GFP construct that was used to survey for green cells and determine successful transfection, which were evident at both 24 and 48 hours post-transfection. At 48 hours post-transfection, whole cell lysates were collected in a total of 1 mL lysis buffer that had been previously used to co-immunoprecipitate MCPyV LT and pRb in 293 cells (50 mM Tris-HCl, 0.15 M NaCl, 1% Triton-X100, pH 7.4; supplemented with protease/phosphatase inhibitors) [42]. Protein concentrations of whole cell lysates were determined using BioRad Protein Assay reagent (BioRad, Hercules, CA). Approximately 500 μg of total protein in a total volume of 1 mL was pre-cleared by incubating with normal rabbit serum (Vector Laboratories; Burlingame, CA) followed by incubation with 40 μL Protein A/G Plus-Agarose (sc-2003) and rotating at 4°C. Beads were removed by centrifugation at 2500 rpm, leaving the pre-cleared lysate (“input”), of which 30 μg was reserved to load as an input control. The remaining pre-cleared lysate was then incubated for 1.5 hours at 4°C with 2 μg anti-pRb antibody (clone H-2; sc-74570; Santa Cruz Biotechnology, Dallas, TX) with constant rotation. To immunoprecipitate immune complexes, 20 μL of Protein A/G Plus-Agarose beads were added to each sample and rotated overnight at 4°C. Beads were collected by centrifugation at 2500 rpm, followed by four washes with lysis buffer. Beads were resuspended in SDS loading buffer, boiled for 5 minutes, resolved using SDS-PAGE electrophoresis with 10% gels poured using TGX Stain-Free FastCast Acrylamide solutions (BioRad; Hercules, CA). Following electrophoresis, immunoblots were performed using the methods detailed below.

Whole tissue lysates were prepared by mincing mouse skin samples that had been snap frozen in liquid nitrogen and stored at -80°C. RIPA lysis buffer supplemented with protease/phosphatase inhibitor cocktail was used for tissue lysis on ice. Lysates were cleared by centrifugation at 14,000 rpm for 30 minutes. Protein concentrations of whole cell or whole tissue lysates were determined using BioRad Protein Assay reagent (BioRad, Hercules, CA). Equivalent concentrations of protein (50 μg skin lysates) were resolved using precast polyacrylamide gels (Mini-Protean TGX AnyKD or Mini-Protean TGX 4–20% gradient gels; BioRad, Hercules, CA). Resolved proteins were then transferred to 0.45 μM nitrocellulose membrane (Whatman Protran BA85; GE Healthcare, Pittsburgh, PA). Following transfer, membranes were stained with 0.1% Ponceau S in 1% acetic acid to verify equal loading and successful transfer of proteins and then blocked with 5% nonfat dry milk in TBS-BGT (Tris-buffered saline containing BSA and glycine supplemented with 0.1% Tween-20). Separated and immobilized proteins were analyzed using the following primary antibodies at the indicated dilutions: β-actin (1:5000; Sigma, St. Louis, MO), COXIV (1:1000; ab33985; Abcam, Cambridge, United Kingdom), MCPyV LT (Ab3) (1:5000;[28, 68]), MCPyV small/LT (Ab5) (1:5000;[28]), and pRb (1:200; sc-74570 clone H-2; Santa Cruz, Dallas, TX). To visualize immune complexes, horseradish peroxidase-conjugated secondary antibodies (1:10,000; Jackson Immunoresearch, West Grove, PA) and chemiluminescent substrate (Clarity ECL; BioRad, Hercules, CA) were applied to membranes, followed by exposure on a ChemiDoc Gel Imaging system (BioRad, Hercules, CA). All washes were performed with TBS-BGT, and blots were stripped with 0.2 M sodium hydroxide where applicable.

To quantify the relative amount of LT immunoprecipitated with pRb, densitometry analysis was performed on immunoblots from three independent co-immunoprecipitation experiments using ImageJ software (NIH, Bethesda, MD). Using images of anti-pRb and anti-LT immunoblots of pRb immunoprecipitates, the density of bands corresponding to immunoprecipitated pRb were quantified from immunoblots from three independent experiments. Likewise, the density of bands corresponding to co-immunoprecipitated LT were quantified from immunoblots from three independent experiments. The average density of immunoprecipitated LT was then normalized to the average density of LT to yield a value for the relative amount of LT co-immunoprecipitated with pRb.

### Immunohistochemistry

Tissue sections were deparaffinized and rehydrated with xylenes and graded ethanol, respectively. For immunohistochemical staining, endogenous peroxidase activity was quenched with 3% H_2_O_2_ in methanol. Heat-induced antigen retrieval was performed in 0.01 M citrate buffer, pH 6.0 solution. Antibodies against the following proteins were used: bromodeoxyuridine BrdU (MilliporeSigma #NA61, Burlington, MA; 1:50), mini-chromosome maintenance protein 7 (MCM7) (MCM7, Thermo Fisher Scientific #MS862, Waltham, MA), biotinylated horse anti-mouse/rabbit IgGs (Vector Laboratories; Burlingame, CA). For immunohistochemical staining, proteins were visualized with 3,3′-diaminobenzidine (DAB) and tissues were counterstained with instant hematoxylin (Vector Laboratories; Burlingame, CA). All images were taken with a Zeiss AxioImager M2 microscope using the AxioVision software version 4.8.2.

### Suprabasal:Basal ratio and BrdU quantitation

The total number of basal and suprabasal cells were quantified using H&E-stained sections of ear tissue and expressed as a suprabasal:basal ratio. The ratio calculated from at least three individual mice from each group was averaged (error bars: ±SD). For BrdU quantitation, one slide from at least three individual mice was analyzed by microscopy and 10 images (20X) were captured. The total number of cells and BrdU-positive cells were quantified with an automated cell counting program [developed by David Ornelles (Wake Forest University School of Medicine, Winston-Salem, NC)] using ImageJ software (NIH, Bethesda, MD)]. This program was developed to allow the user to manually choose a region of interest on an image of a stained tissue section, within which the total number of cells and BrdU-positive cells (brown nuclei) are quantified. This program has been compared to manual quantitation and yields results indistinguishable to those counted by hand (S1 Fig). The percentage of total BrdU-positive cells was calculated for each sample using the average of 10 fields from each mouse. These values were then averaged among the mice in each group. The standard deviation reflects variation between individual mice. A two-sided Wilcoxon rank sum test was used to compare the average percentage of BrdU-positive cells between groups. Statistical analysis was performed using MSTAT software (version 7.0 last accessed September 27, 2021). Graphs were generated using GraphPad Prism (Version 9.1.2). For all analyses, tissue sections from both white and brown offspring were included.

### E2F luciferase assays and quantitation

Primary mouse embryonic fibroblasts (MEFs) were generated from wild-type (RbWT) and *Rb1*^*ΔL/ΔL*^ (RbΔL) matched littermates as described previously [55, 59]. MEFs were cultured in DMEM supplemented with 10% fetal bovine serum (FBS), 2 mM l-glutamine, penicillin (50 U/ml), and streptomycin (50 μg/ml). All experiments were carried out using MEFs at passage 4 from Rb^WT^ and Rb^ΔL^ matched littermates from two different litters.

To perform E2F luciferase experiments, MEFs were plated in triplicate at a density of 3 x 10^4^ cells/well in a Nunc 96-well white clear-bottom plate (Thermo Scientific; # 165306) in culture media without antibiotics. Approximately 2 hours post-plating, cells were transfected with Lipofectamine 2000 Transfection Reagent according to the manufacturer protocol (Thermo Fisher; Waltham, MA). All cells were transfected with 0.4 μL Lipofectamine 2000 reagent and a total of 100 ng DNA/well: 20 ng *Renilla* luciferase plasmid pRL-TK (Promega, Madison, WI, #E2241) used as a control for transfection efficiency, 40 ng of the E2F luciferase reporter (pGL3-6XE2F; kindly provided by Dr. Karl Munger (Tufts University) and described previously [58]), and 40 ng of viral effector protein. In the reporter-only condition where no viral effector protein was transfected, pcDNA6-dsRed was used to keep the total amount of transfected DNA constant and to survey for red cells as a visual readout for successful transfection, which were evident at both 24 and 48 hours post-transfection in all MEF strains. The following constructs were used to express viral effector proteins: pLenti6.3-MCPyV168ER (described above), pLXSN-HPV16E7 (kindly provided by Dr. Karl Munger (Tufts University) and described previously [69]), and pCMV-TAg to express SV40 LT [70]. At 48 hours post-transfection, a dual luciferase assay was performed using the Dual-Glo Luciferase Assay kit (Promega, Madison, WI; #E2920). Luminescent signals were read using a BMG Pherastar Multimode plate reader (BMG Labtech, Cary, NC) at the Small Molecule Screening Facility (University of Wisconsin Carbone Cancer Center, UW-Madison). Data was analyzed by normalizing firefly luciferase values to *Renilla* luciferase values for all conditions. The reporter + viral effector conditions were normalized to reporter only (no viral effector) to generate the graph in Fig 3D.

## Supporting information

S1 Fig Quantitation of total and BrdU-positive cells in stratified epithelia. **A)** The major steps during quantitation of total and BrdU-positive cells from representative images of BrdU immunohistochemistry using an automated counting program in ImageJ. First, a region of interest (ROI) is defined by the user. The program then quantifies the total number of cells within the ROI. The total number of BrdU-positive cells, as defined by brown nuclei, are then quantified. **B)** Comparison of the total percentage of BrdU-positive cells quantified using manual counting (black bars) and automated counting (gray bars) in three different groups of mice (n = 3 each). Error bars indicate standard deviation.
(TIF)

## References

[1] Harms PW, Harms KL, Moore PS, DeCaprio JA, Nghiem P, Wong MKK, et al. The biology and treatment of Merkel cell carcinoma: current understanding and research priorities. Nat Rev Clin Oncol. 2018;15(12):763–76. Epub 2018/10/06. doi: 10.1038/s41571-018-0103-2 ; PubMed Central PMCID: PMC6319370. 30287935 PMC6319370

[2] Heath M, Jaimes N, Lemos B, Mostaghimi A, Wang LC, Penas PF, et al. Clinical characteristics of Merkel cell carcinoma at diagnosis in 195 patients: the AEIOU features. J Am Acad Dermatol. 2008;58(3):375–81. Epub 2008/02/19. doi: 10.1016/j.jaad.2007.11.020 ; PubMed Central PMCID: PMC2335370. 18280333 PMC2335370

[3] Hodgson NC. Merkel cell carcinoma: changing incidence trends. J Surg Oncol. 2005;89(1):1–4. Epub 2004/12/22. doi: 10.1002/jso.20167 . 15611998

[4] Paulson KG, Park SY, Vandeven NA, Lachance K, Thomas H, Chapuis AG, et al. Merkel cell carcinoma: Current US incidence and projected increases based on changing demographics. J Am Acad Dermatol. 2018;78(3):457–63 e2. Epub 2017/11/06. doi: 10.1016/j.jaad.2017.10.028 ; PubMed Central PMCID: PMC5815902. 29102486 PMC5815902

[5] Feng H, Shuda M, Chang Y, Moore PS. Clonal integration of a polyomavirus in human Merkel cell carcinoma. Science. 2008;319(5866):1096–100. Epub 2008/01/19. doi: 10.1126/science.1152586 ; PubMed Central PMCID: PMC2740911. 18202256 PMC2740911

[6] DeCaprio JA, Garcea RL. A cornucopia of human polyomaviruses. Nat Rev Microbiol. 2013;11(4):264–76. Epub 2013/03/12. doi: 10.1038/nrmicro2992 ; PubMed Central PMCID: PMC3928796. 23474680 PMC3928796

[7] Spurgeon ME, Lambert PF. Merkel cell polyomavirus: a newly discovered human virus with oncogenic potential. Virology. 2013;435(1):118–30. Epub 2012/12/12. doi: 10.1016/j.virol.2012.09.029 ; PubMed Central PMCID: PMC3522868. 23217622 PMC3522868

[8] Carter JJ, Paulson KG, Wipf GC, Miranda D, Madeleine MM, Johnson LG, et al. Association of Merkel cell polyomavirus-specific antibodies with Merkel cell carcinoma. J Natl Cancer Inst. 2009;101(21):1510–22. Epub 2009/09/25. doi: 10.1093/jnci/djp332 ; PubMed Central PMCID: PMC2773184. 19776382 PMC2773184

[9] Chen T, Hedman L, Mattila PS, Jartti T, Ruuskanen O, Soderlund-Venermo M, et al. Serological evidence of Merkel cell polyomavirus primary infections in childhood. J Clin Virol. 2011;50(2):125–9. Epub 2010/11/26. doi: 10.1016/j.jcv.2010.10.015 . 21094082

[10] Kean JM, Rao S, Wang M, Garcea RL. Seroepidemiology of human polyomaviruses. PLoS Pathog. 2009;5(3):e1000363. Epub 2009/03/28. doi: 10.1371/journal.ppat.1000363 ; PubMed Central PMCID: PMC2655709. 19325891 PMC2655709

[11] Pastrana DV, Tolstov YL, Becker JC, Moore PS, Chang Y, Buck CB. Quantitation of human seroresponsiveness to Merkel cell polyomavirus. PLoS Pathog. 2009;5(9):e1000578. Epub 2009/09/15. doi: 10.1371/journal.ppat.1000578 ; PubMed Central PMCID: PMC2734180. 19750217 PMC2734180

[12] Tolstov YL, Knauer A, Chen JG, Kensler TW, Kingsley LA, Moore PS, et al. Asymptomatic primary Merkel cell polyomavirus infection among adults. Emerg Infect Dis. 2011;17(8):1371–80. Epub 2011/08/02. doi: 10.3201/eid1708.110079 ; PubMed Central PMCID: PMC3381535. 21801612 PMC3381535

[13] Tolstov YL, Pastrana DV, Feng H, Becker JC, Jenkins FJ, Moschos S, et al. Human Merkel cell polyomavirus infection II. MCV is a common human infection that can be detected by conformational capsid epitope immunoassays. Int J Cancer. 2009;125(6):1250–6. Epub 2009/06/06. doi: 10.1002/ijc.24509 ; PubMed Central PMCID: PMC2747737. 19499548 PMC2747737

[14] Touze A, Gaitan J, Arnold F, Cazal R, Fleury MJ, Combelas N, et al. Generation of Merkel cell polyomavirus (MCV)-like particles and their application to detection of MCV antibodies. J Clin Microbiol. 2010;48(5):1767–70. Epub 2010/02/26. doi: 10.1128/JCM.01691-09 ; PubMed Central PMCID: PMC2863896. 20181914 PMC2863896

[15] Viscidi RP, Rollison DE, Sondak VK, Silver B, Messina JL, Giuliano AR, et al. Age-specific seroprevalence of Merkel cell polyomavirus, BK virus, and JC virus. Clin Vaccine Immunol. 2011;18(10):1737–43. Epub 2011/09/02. doi: 10.1128/CVI.05175-11 ; PubMed Central PMCID: PMC3187023. 21880855 PMC3187023

[16] Schowalter RM, Pastrana DV, Pumphrey KA, Moyer AL, Buck CB. Merkel cell polyomavirus and two previously unknown polyomaviruses are chronically shed from human skin. 2010;7(6):509–15. Epub 2010/06/15. doi: 10.1016/j.chom.2010.05.006 ; PubMed Central PMCID: PMC2919322. 20542254 PMC2919322

[17] Becker M, Dominguez M, Greune L, Soria-Martinez L, Pfleiderer MM, Schowalter R, et al. Infectious Entry of Merkel Cell Polyomavirus. J Virol. 2019;93(6). Epub 2019/01/11. doi: 10.1128/JVI.02004-18 ; PubMed Central PMCID: PMC6401430. 30626687 PMC6401430

[18] Kwun HJ, Chang Y, Moore PS. Protein-mediated viral latency is a novel mechanism for Merkel cell polyomavirus persistence. Proc Natl Acad Sci U S A. 2017;114(20):E4040–E7. Epub 2017/05/04. doi: 10.1073/pnas.1703879114 ; PubMed Central PMCID: PMC5441811. 28461484 PMC5441811

[19] Liu W, Krump NA, Buck CB, You J. Merkel Cell Polyomavirus Infection and Detection. J Vis Exp. 2019;(144). Epub 2019/02/26. doi: 10.3791/58950 ; PubMed Central PMCID: PMC6656558. 30799855 PMC6656558

[20] Neumann F, Borchert S, Schmidt C, Reimer R, Hohenberg H, Fischer N, et al. Replication, gene expression and particle production by a consensus Merkel Cell Polyomavirus (MCPyV) genome. PLoS One. 2011;6(12):e29112. Epub 2012/01/05. doi: 10.1371/journal.pone.0029112 ; PubMed Central PMCID: PMC3246459. 22216177 PMC3246459

[21] Schowalter RM, Reinhold WC, Buck CB. Entry tropism of BK and Merkel cell polyomaviruses in cell culture. PLoS One. 2012;7(7):e42181. Epub 2012/08/04. doi: 10.1371/journal.pone.0042181 ; PubMed Central PMCID: PMC3409148. 22860078 PMC3409148

[22] DeCaprio JA. Merkel cell polyomavirus and Merkel cell carcinoma. Philos Trans R Soc Lond B Biol Sci. 2017;372(1732). Epub 2017/09/13. doi: 10.1098/rstb.2016.0276 ; PubMed Central PMCID: PMC5597743. 28893943 PMC5597743

[23] Krump NA, You J. From Merkel Cell Polyomavirus Infection to Merkel Cell Carcinoma Oncogenesis. Front Microbiol. 2021;12:739695. Epub 2021/09/28. doi: 10.3389/fmicb.2021.739695 ; PubMed Central PMCID: PMC8457551. 34566942 PMC8457551

[24] Spurgeon ME, Liem A, Buehler D, Cheng J, DeCaprio JA, Lambert PF. The Merkel Cell Polyomavirus T Antigens Function as Tumor Promoters in Murine Skin. Cancers (Basel). 2021;13(2). Epub 2021/01/14. doi: 10.3390/cancers13020222 ; PubMed Central PMCID: PMC7827793. 33435392 PMC7827793

[25] Angermeyer S, Hesbacher S, Becker JC, Schrama D, Houben R. Merkel cell polyomavirus-positive Merkel cell carcinoma cells do not require expression of the viral small T antigen. J Invest Dermatol. 2013;133(8):2059–64. Epub 2013/02/27. doi: 10.1038/jid.2013.82 . 23439392

[26] Houben R, Shuda M, Weinkam R, Schrama D, Feng H, Chang Y, et al. Merkel cell polyomavirus-infected Merkel cell carcinoma cells require expression of viral T antigens. J Virol. 2010;84(14):7064–72. Epub 2010/05/07. doi: 10.1128/JVI.02400-09 ; PubMed Central PMCID: PMC2898224. 20444890 PMC2898224

[27] Shuda M, Chang Y, Moore PS. Merkel cell polyomavirus-positive Merkel cell carcinoma requires viral small T-antigen for cell proliferation. J Invest Dermatol. 2014;134(5):1479–81. Epub 2013/11/13. doi: 10.1038/jid.2013.483 ; PubMed Central PMCID: PMC3989379. 24217011 PMC3989379

[28] Cheng J, Rozenblatt-Rosen O, Paulson KG, Nghiem P, DeCaprio JA. Merkel cell polyomavirus large T antigen has growth-promoting and inhibitory activities. J Virol. 2013;87(11):6118–26. Epub 2013/03/22. doi: 10.1128/JVI.00385-13 ; PubMed Central PMCID: PMC3648111. 23514892 PMC3648111

[29] Nwogu N, Ortiz LE, Kwun HJ. Surface charge of Merkel cell polyomavirus small T antigen determines cell transformation through allosteric FBW7 WD40 domain targeting. Oncogenesis. 2020;9(5):53. Epub 2020/05/20. doi: 10.1038/s41389-020-0235-y ; PubMed Central PMCID: PMC7237485. 32427880 PMC7237485

[30] Shuda M, Kwun HJ, Feng H, Chang Y, Moore PS. Human Merkel cell polyomavirus small T antigen is an oncoprotein targeting the 4E-BP1 translation regulator. J Clin Invest. 2011;121(9):3623–34. Epub 2011/08/16. doi: 10.1172/JCI46323 ; PubMed Central PMCID: PMC3163959. 21841310 PMC3163959

[31] Shuda M, Guastafierro A, Geng X, Shuda Y, Ostrowski SM, Lukianov S, et al. Merkel Cell Polyomavirus Small T Antigen Induces Cancer and Embryonic Merkel Cell Proliferation in a Transgenic Mouse Model. PLoS One. 2015;10(11):e0142329. Epub 2015/11/07. doi: 10.1371/journal.pone.0142329 ; PubMed Central PMCID: PMC4636375. 26544690 PMC4636375

[32] Velasquez C, Cheng E, Shuda M, Lee-Oesterreich PJ, Pogge von Strandmann L, Gritsenko MA, et al. Mitotic protein kinase CDK1 phosphorylation of mRNA translation regulator 4E-BP1 Ser83 may contribute to cell transformation. Proc Natl Acad Sci U S A. 2016;113(30):8466–71. Epub 2016/07/13. doi: 10.1073/pnas.1607768113 ; PubMed Central PMCID: PMC4968757. 27402756 PMC4968757

[33] Wu JH, Simonette RA, Hsiao T, Doan HQ, Rady PL, Tyring SK. Cutaneous Human Polyomavirus Small T Antigens and 4E-BP1 Targeting. Intervirology. 2015;58(6):382–5. Epub 2016/04/08. doi: 10.1159/000444921 . 27055259

[34] Cheng J, Park DE, Berrios C, White EA, Arora R, Yoon R, et al. Merkel cell polyomavirus recruits MYCL to the EP400 complex to promote oncogenesis. PLoS Pathog. 2017;13(10):e1006668. Epub 2017/10/14. doi: 10.1371/journal.ppat.1006668 ; PubMed Central PMCID: PMC5640240. 29028833 PMC5640240

[35] Verhaegen ME, Harms PW, Van Goor JJ, Arche J, Patrick MT, Wilbert D, et al. Direct cellular reprogramming enables development of viral T antigen-driven Merkel cell carcinoma in mice. J Clin Invest. 2022;132(7). Epub 2022/02/11. doi: 10.1172/JCI152069 ; PubMed Central PMCID: PMC8970662. 35143422 PMC8970662

[36] Verhaegen ME, Mangelberger D, Harms PW, Eberl M, Wilbert DM, Meireles J, et al. Merkel Cell Polyomavirus Small T Antigen Initiates Merkel Cell Carcinoma-like Tumor Development in Mice. Cancer Res. 2017;77(12):3151–7. Epub 2017/05/18. doi: 10.1158/0008-5472.CAN-17-0035 ; PubMed Central PMCID: PMC5635997. 28512245 PMC5635997

[37] Verhaegen ME, Mangelberger D, Harms PW, Vozheiko TD, Weick JW, Wilbert DM, et al. Merkel cell polyomavirus small T antigen is oncogenic in transgenic mice. J Invest Dermatol. 2015;135(5):1415–24. Epub 2014/10/15. doi: 10.1038/jid.2014.446 ; PubMed Central PMCID: PMC4397111. 25313532 PMC4397111

[38] Hesbacher S, Pfitzer L, Wiedorfer K, Angermeyer S, Borst A, Haferkamp S, et al. RB1 is the crucial target of the Merkel cell polyomavirus Large T antigen in Merkel cell carcinoma cells. Oncotarget. 2016;7(22):32956–68. Epub 2016/04/29. doi: 10.18632/oncotarget.8793 ; PubMed Central PMCID: PMC5078066. 27121059 PMC5078066

[39] Houben R, Adam C, Baeurle A, Hesbacher S, Grimm J, Angermeyer S, et al. An intact retinoblastoma protein-binding site in Merkel cell polyomavirus large T antigen is required for promoting growth of Merkel cell carcinoma cells. Int J Cancer. 2012;130(4):847–56. Epub 2011/03/18. doi: 10.1002/ijc.26076 . 21413015

[40] Schmitt M, Wieland U, Kreuter A, Pawlita M. C-terminal deletions of Merkel cell polyomavirus large T-antigen, a highly specific surrogate marker for virally induced malignancy. Int J Cancer. 2012;131(12):2863–8. Epub 2012/06/08. doi: 10.1002/ijc.27607 . 22674148

[41] Schrama D, Sarosi EM, Adam C, Ritter C, Kaemmerer U, Klopocki E, et al. Characterization of six Merkel cell polyomavirus-positive Merkel cell carcinoma cell lines: Integration pattern suggest that large T antigen truncating events occur before or during integration. Int J Cancer. 2019;145(4):1020–32. Epub 2019/03/16. doi: 10.1002/ijc.32280 . 30873613

[42] Shuda M, Feng H, Kwun HJ, Rosen ST, Gjoerup O, Moore PS, et al. T antigen mutations are a human tumor-specific signature for Merkel cell polyomavirus. Proc Natl Acad Sci U S A. 2008;105(42):16272–7. Epub 2008/09/25. doi: 10.1073/pnas.0806526105 ; PubMed Central PMCID: PMC2551627. 18812503 PMC2551627

[43] Borchert S, Czech-Sioli M, Neumann F, Schmidt C, Wimmer P, Dobner T, et al. High-affinity Rb binding, p53 inhibition, subcellular localization, and transformation by wild-type or tumor-derived shortened Merkel cell polyomavirus large T antigens. J Virol. 2014;88(6):3144–60. Epub 2013/12/29. doi: 10.1128/JVI.02916-13 ; PubMed Central PMCID: PMC3957953. 24371076 PMC3957953

[44] Lambert PF. The interwoven story of the small DNA tumor viruses. Virology. 2009;384(2):255. Epub 2009/02/11. doi: 10.1016/j.virol.2008.12.008 . 19203639

[45] Schrama D, Hesbacher S, Angermeyer S, Schlosser A, Haferkamp S, Aue A, et al. Serine 220 phosphorylation of the Merkel cell polyomavirus large T antigen crucially supports growth of Merkel cell carcinoma cells. Int J Cancer. 2016;138(5):1153–62. Epub 2015/09/19. doi: 10.1002/ijc.29862 . 26383606

[46] Sihto H, Kukko H, Koljonen V, Sankila R, Bohling T, Joensuu H. Merkel cell polyomavirus infection, large T antigen, retinoblastoma protein and outcome in Merkel cell carcinoma. Clin Cancer Res. 2011;17(14):4806–13. Epub 2011/06/07. doi: 10.1158/1078-0432.CCR-10-3363 . 21642382

[47] Richards KF, Guastafierro A, Shuda M, Toptan T, Moore PS, Chang Y. Merkel cell polyomavirus T antigens promote cell proliferation and inflammatory cytokine gene expression. J Gen Virol. 2015;96(12):3532–44. Epub 2015/09/20. doi: 10.1099/jgv.0.000287 ; PubMed Central PMCID: PMC4804762. 26385761 PMC4804762

[48] Starrett GJ, Marcelus C, Cantalupo PG, Katz JP, Cheng J, Akagi K, et al. Merkel Cell Polyomavirus Exhibits Dominant Control of the Tumor Genome and Transcriptome in Virus-Associated Merkel Cell Carcinoma. mBio. 2017;8(1). Epub 2017/01/04. doi: 10.1128/mBio.02079-16 ; PubMed Central PMCID: PMC5210499. 28049147 PMC5210499

[49] Park DE, Cheng J, Berrios C, Montero J, Cortes-Cros M, Ferretti S, et al. Dual inhibition of MDM2 and MDM4 in virus-positive Merkel cell carcinoma enhances the p53 response. Proc Natl Acad Sci U S A. 2019;116(3):1027–32. Epub 2019/01/02. doi: 10.1073/pnas.1818798116 ; PubMed Central PMCID: PMC6338866. 30598450 PMC6338866

[50] Fan K, Gravemeyer J, Ritter C, Rasheed K, Gambichler T, Moens U, et al. MCPyV Large T Antigen-Induced Atonal Homolog 1 Is a Lineage-Dependency Oncogene in Merkel Cell Carcinoma. J Invest Dermatol. 2020;140(1):56–65 e3. Epub 2019/07/10. doi: 10.1016/j.jid.2019.06.135 . 31283928

[51] Harold A, Amako Y, Hachisuka J, Bai Y, Li MY, Kubat L, et al. Conversion of Sox2-dependent Merkel cell carcinoma to a differentiated neuron-like phenotype by T antigen inhibition. Proc Natl Acad Sci U S A. 2019;116(40):20104–14. Epub 2019/09/19. doi: 10.1073/pnas.1907154116 ; PubMed Central PMCID: PMC6778204. 31527246 PMC6778204

[52] Spurgeon ME, Cheng J, Bronson RT, Lambert PF, DeCaprio JA. Tumorigenic activity of merkel cell polyomavirus T antigens expressed in the stratified epithelium of mice. Cancer Res. 2015;75(6):1068–79. Epub 2015/01/18. doi: 10.1158/0008-5472.CAN-14-2425 ; PubMed Central PMCID: PMC4359959. 25596282 PMC4359959

[53] Dick FA, Dyson NJ. Three regions of the pRB pocket domain affect its inactivation by human papillomavirus E7 proteins. J Virol. 2002;76(12):6224–34. Epub 2002/05/22. doi: 10.1128/jvi.76.12.6224-6234.2002 ; PubMed Central PMCID: PMC136242. 12021356 PMC136242

[54] Dick FA, Sailhamer E, Dyson NJ. Mutagenesis of the pRB pocket reveals that cell cycle arrest functions are separable from binding to viral oncoproteins. Mol Cell Biol. 2000;20(10):3715–27. Epub 2000/04/25. doi: 10.1128/MCB.20.10.3715-3727.2000 ; PubMed Central PMCID: PMC85672. 10779361 PMC85672

[55] Isaac CE, Francis SM, Martens AL, Julian LM, Seifried LA, Erdmann N, et al. The retinoblastoma protein regulates pericentric heterochromatin. Mol Cell Biol. 2006;26(9):3659–71. Epub 2006/04/14. doi: 10.1128/MCB.26.9.3659-3671.2006 ; PubMed Central PMCID: PMC1447412. 16612004 PMC1447412

[56] Balsitis S, Dick F, Dyson N, Lambert PF. Critical roles for non-pRb targets of human papillomavirus type 16 E7 in cervical carcinogenesis. Cancer Res. 2006;66(19):9393–400. Epub 2006/10/05. doi: 10.1158/0008-5472.CAN-06-0984 ; PubMed Central PMCID: PMC2858286. 17018593 PMC2858286

[57] Balsitis S, Dick F, Lee D, Farrell L, Hyde RK, Griep AE, et al. Examination of the pRb-dependent and pRb-independent functions of E7 in vivo. J Virol. 2005;79(17):11392–402. Epub 2005/08/17. doi: 10.1128/JVI.79.17.11392-11402.2005 ; PubMed Central PMCID: PMC1193607. 16103190 PMC1193607

[58] Muller H, Moroni MC, Vigo E, Petersen BO, Bartek J, Helin K. Induction of S-phase entry by E2F transcription factors depends on their nuclear localization. Mol Cell Biol. 1997;17(9):5508–20. Epub 1997/09/01. doi: 10.1128/MCB.17.9.5508 ; PubMed Central PMCID: PMC232399. 9271426 PMC232399

[59] Hurford RK Jr., Cobrinik D, Lee MH, Dyson N. pRB and p107/p130 are required for the regulated expression of different sets of E2F responsive genes. Genes Dev. 1997;11(11):1447–63. Epub 1997/06/01. doi: 10.1101/gad.11.11.1447 9192872

[60] Grace M, Munger K. Proteomic analysis of the gamma human papillomavirus type 197 E6 and E7 associated cellular proteins. Virology. 2017;500:71–81. Epub 2016/10/25. doi: 10.1016/j.virol.2016.10.010 ; PubMed Central PMCID: PMC5127743. 27771561 PMC5127743

[61] McLaughlin-Drubin ME, Huh KW, Munger K. Human papillomavirus type 16 E7 oncoprotein associates with E2F6. J Virol. 2008;82(17):8695–705. Epub 2008/06/27. doi: 10.1128/JVI.00579-08 ; PubMed Central PMCID: PMC2519642. 18579589 PMC2519642

[62] Wakabayashi Y, Mao JH, Brown K, Girardi M, Balmain A. Promotion of Hras-induced squamous carcinomas by a polymorphic variant of the Patched gene in FVB mice. Nature. 2007;445(7129):761–5. Epub 2007/01/19. doi: 10.1038/nature05489 . 17230190

[63] Brown VD, Gallie BL. The B-domain lysine patch of pRB is required for binding to large T antigen and release of E2F by phosphorylation. Mol Cell Biol. 2002;22(5):1390–401. Epub 2002/02/13. doi: 10.1128/MCB.22.5.1390-1401.2002 ; PubMed Central PMCID: PMC134706. 11839806 PMC134706

[64] Czech-Sioli M, Siebels S, Radau S, Zahedi RP, Schmidt C, Dobner T, et al. The Ubiquitin-Specific Protease Usp7, a Novel Merkel Cell Polyomavirus Large T-Antigen Interaction Partner, Modulates Viral DNA Replication. J Virol. 2020;94(5). Epub 2019/12/06. doi: 10.1128/JVI.01638-19 ; PubMed Central PMCID: PMC7022344. 31801860 PMC7022344

[65] Liu X, Hein J, Richardson SC, Basse PH, Toptan T, Moore PS, et al. Merkel cell polyomavirus large T antigen disrupts lysosome clustering by translocating human Vam6p from the cytoplasm to the nucleus. J Biol Chem. 2011;286(19):17079–90. Epub 2011/04/02. doi: 10.1074/jbc.M110.192856 ; PubMed Central PMCID: PMC3089552. 21454559 PMC3089552

[66] Houben R, Angermeyer S, Haferkamp S, Aue A, Goebeler M, Schrama D, et al. Characterization of functional domains in the Merkel cell polyoma virus Large T antigen. Int J Cancer. 2015;136(5):E290–300. Epub 2014/09/12. doi: 10.1002/ijc.29200 . 25208506

[67] Kervarrec T, Samimi M, Hesbacher S, Berthon P, Wobser M, Sallot A, et al. Merkel Cell Polyomavirus T Antigens Induce Merkel Cell-Like Differentiation in GLI1-Expressing Epithelial Cells. Cancers (Basel). 2020;12(7). Epub 2020/07/28. doi: 10.3390/cancers12071989 ; PubMed Central PMCID: PMC7409360. 32708246 PMC7409360

[68] Rodig SJ, Cheng J, Wardzala J, DoRosario A, Scanlon JJ, Laga AC, et al. Improved detection suggests all Merkel cell carcinomas harbor Merkel polyomavirus. J Clin Invest. 2012;122(12):4645–53. Epub 2012/11/02. doi: 10.1172/JCI64116 ; PubMed Central PMCID: PMC3533549. 23114601 PMC3533549

[69] Halbert CL, Demers GW, Galloway DA. The E7 gene of human papillomavirus type 16 is sufficient for immortalization of human epithelial cells. J Virol. 1991;65(1):473–8. Epub 1991/01/01. doi: 10.1128/JVI.65.1.473-478.1991 ; PubMed Central PMCID: PMC240541. 1845902 PMC240541

[70] Campbell KS, Mullane KP, Aksoy IA, Stubdal H, Zalvide J, Pipas JM, et al. DnaJ/hsp40 chaperone domain of SV40 large T antigen promotes efficient viral DNA replication. Genes Dev. 1997;11(9):1098–110. Epub 1997/05/01. doi: 10.1101/gad.11.9.1098 . 9159391

