# Supplementary figures and images for: Merkel cell polyomavirus large T antigen binding to pRb promotes skin hyperplasia and tumor development
Source: PLoS Pathog. 2022 May 13;18(5):e1010551. doi: 10.1371/journal.ppat.1010551 (PMC9132321; doi:10.1371/journal.ppat.1010551)

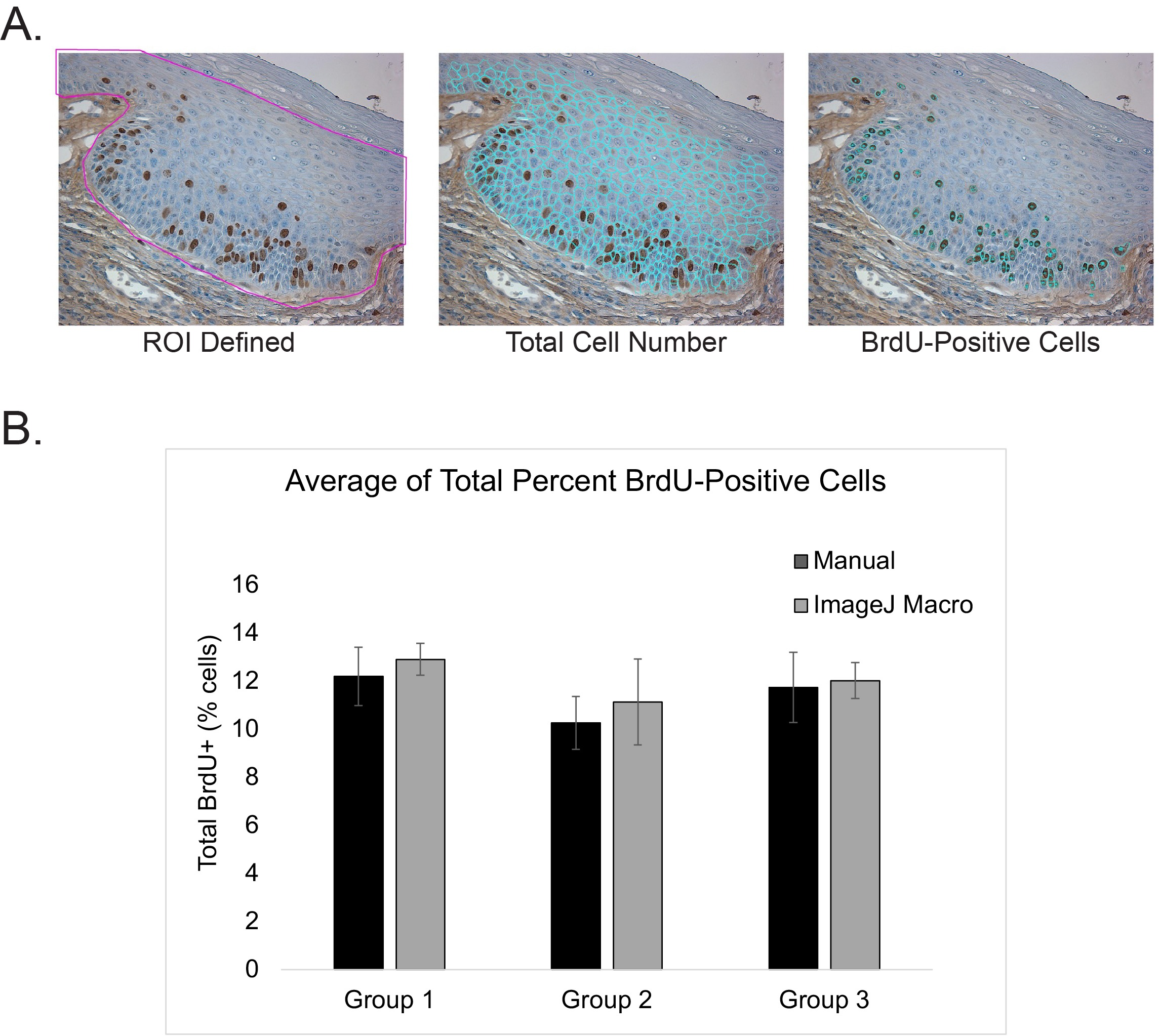

Supplement: S1 Fig — A) The major steps during quantitation of total and BrdU-positive cells from representative images of BrdU immunohistochemistry using an automated counting program in ImageJ. First, a region of interest (ROI) is defined by the user. The program then quantifies the total number of cells within the ROI. The total number of BrdU-positive cells, as defined by brown nuclei, are then quantified. B) Comparison of the total percentage of BrdU-positive cells quantified using manual counting (black bars) and automated counting (gray bars) in three different groups of mice (n = 3 each). Error bars indicate standard deviation. (TIF) [file ppat.1010551.s001.tif]
